# Improved grain mapping by laboratory X-ray diffraction contrast tomography

**DOI:** 10.1107/S2052252521003730

**Published:** 2021-05-07

**Authors:** H. Fang, D. Juul Jensen, Y. Zhang

**Affiliations:** a Department of Mechanical Engineering, Technical University of Denmark, Kgs. Lyngby, 2800, Denmark

**Keywords:** 3D grain mapping, diffraction contrast tomography, X-ray diffraction, spatial resolution, magnified geometries, detection limits

## Abstract

Laboratory diffraction contrast tomography (LabDCT) is a novel technique for non-destructive imaging of the grain structure within polycrystalline samples. To further broaden the use of this technique to a wider range of materials, both the spatial resolution and detection limit achieved in the commonly used Laue focusing geometry have to be improved. In this work, the possibility of improving both grain indexing and shape reconstruction was investigated by increasing the sample-to-detector distance to facilitate geometrical magnification of diffraction spots in the LabDCT projections. LabDCT grain reconstructions of a fully recrystallized iron sample, obtained in the conventional Laue focusing geometry and in a magnified geometry, are compared to one characterized by synchrotron X-ray diffraction contrast tomography, with the latter serving as the ground truth. It is shown that grain indexing can be significantly improved in the magnified geometry. It is also found that the magnified geometry improves the spatial resolution and the accuracy of the reconstructed grain shapes. The improvement is shown to be more evident for grains smaller than 40 µm than for larger grains. The underlying reasons are clarified by comparing spot features for different LabDCT datasets using a forward simulation tool.

## Abstract 2

The present laboratory diffraction contrast tomography suffers from poor spatial resolution – only grains larger than 40 µm can be reliably imaged. In this work, it is shown how this serious limitation can be lifted and the spatial resolution improved significantly.

## 1. Introduction   

In the last two decades non-destructive 3D characterization of grain sizes, shapes and orientations within the bulk of crystalline materials has come into focus (for an overview see *e.g.* Juul Jensen & Zhang, 2020 ▸). Several techniques have been developed at third-generation synchrotron X-ray facilities for such characterization. These include differential aperture X-ray microscopy (Larson *et al.*, 2002 ▸), 3D X-ray diffraction microscopy (Poulsen *et al.*, 2001 ▸; Poulsen, 2004 ▸, 2012 ▸) and its variants like high-energy X-ray diffraction microscopy (Suter *et al.*, 2006 ▸), scanning 3D X-ray diffraction microscopy (Hayashi *et al.*, 2015 ▸; Henningsson *et al.*, 2020 ▸) and diffraction contrast tomography (DCT) (Ludwig *et al.*, 2008 ▸; Reischig *et al.*, 2013 ▸; Reischig & Ludwig, 2020 ▸), as well as dark-field X-ray microscopy (Simons *et al.*, 2015 ▸; Jakobsen *et al.*, 2019 ▸; Poulsen, 2020 ▸). These methods allow grain mapping at different length scales.

To broaden the use of non-destructive grain mapping by offering such possibilities at home laboratories, laboratory X-ray diffraction contrast tomography (LabDCT) has been developed based on inspirations from synchrotron radiation X-ray diffraction contrast tomography (SR-DCT) (King *et al.*, 2013 ▸, 2014 ▸; McDonald *et al.*, 2015 ▸). Thereby, daily access is assured as opposed to similar measurement at synchrotron sources where only a few experiments can be carried out per year, in the best case. LabDCT has already become a very useful tool to study grain structures in 3D as well as structural evolutions in 4D (space and time) (McDonald *et al.*, 2017 ▸; Pankhurst *et al.*, 2019 ▸; Sun *et al.*, 2019 ▸, 2020 ▸; Lei *et al.*, 2021 ▸).

LabDCT adopts a cone-shaped polychromatic X-ray beam generated from a conventional X-ray tube to illuminate a sample with a typical size of hundreds of micrometres to millimetres. An aperture is normally placed close to the source to confine the direct X-ray beam. X-rays diffracted from the sample in transmission geometry are recorded by a 2D detector, while the direct transmitted beam is shielded by a beamstop to enhance the diffraction signal. A complete dataset for grain reconstruction is acquired by recording diffraction patterns at preset intervals of a 360° sample rotation around a vertical axis. This laboratory-based grain-mapping technique has been commercialized by adding the LabDCT module (with an aperture close to the source and a beamstop in front of the detector) onto a conventional tomography system, *e.g.* Zeiss Xradia 520 Versa. The Laue focusing geometry, where the sample-to-detector distance (*L*_sd_) equals the sample-to-source distance (*L*_ss_), is applied in the current use of the commercial LabDCT. Thereby, the diffraction spots become focused into line features. It is generally assumed that this focusing results in less spot overlap and an increase in signal-to-noise ratio (SNR) (Bachmann *et al.*, 2019 ▸). However, this assumption has not been validated. Additionally, the Laue focusing geometry allows no geometrical magnification of diffraction spots, which may be expected to provide more information on the grain shapes (GSs) and thus a possibility to improve the spatial resolution, particularly for small grains. The large potentials gained by geometrical magnification of diffraction spots that can be easily realized by increasing the ratio of *L*_sd_/*L*_ss_ seem to be evident (King *et al.*, 2013 ▸). As reported by King *et al.* (2013 ▸), the earlier work on LabDCT (before its commercialization) was implemented in a large magnified geometry with *L*_sd_/*L*_ss_ ≃ 24 using a relatively low-resolution detector (1920 × 1536 pixels with a pixel size of 0.127 mm), resulting in a maximum accessible scattering angle of ∼15.7°. However, such a detector system is not suitable for Laue focusing geometries and thus no comparison has been made between results obtained with the magnified geometry and the Laue focusing geometry. It is also yet to be studied how geometrical magnification affects the grain indexing and shape reconstruction. This is particularly interesting as on the one hand the spots are magnified but on the other hand their sharpness is expected to deteriorate.

The aim of the present work is to quantify the effects of increasing *L*_sd_/*L*_ss_ on grain mapping using a commercial LabDCT setup (with a fixed source, a fixed detector system and geometrical constraints in the choice of source-to-detector distances). This is carried out by comparing two grain structures reconstructed from LabDCT diffraction projections: one measured in the Laue focusing geometry (*L*_sd_ = *L*_ss_) and the other in a magnified geometry (*L*_sd_/*L*_ss_ = 1.64). A suitable ground-truth grain structure, which can be used as a reference, is needed to validate the results. Here, we use the grain structure obtained by SR-DCT because it has a better spatial resolution and detection limit than the current LabDCT. As validated by other techniques including phase-contrast tomography (Reischig *et al.*, 2013 ▸), high-energy X-ray microscopy (Renversade *et al.*, 2016 ▸) and electron-backscatter-diffraction mapping (Johnson *et al.*, 2008 ▸; Syha *et al.*, 2013 ▸), SR-DCT has an orientation resolution of <0.1°, a detection limit of ∼5 µm (smallest detectable grain in terms of equivalent spherical diameter) and a spatial resolution [accuracy of grain boundary (GB) position] of ∼1.5 µm for fully recrystallized samples. It was also found that SR-DCT was able to characterize grains of >10 µm in a partially recrystallized Al sample (Sun *et al.*, 2018 ▸). Compared with SR-DCT, LabDCT performs less well, except for a similar orientation resolution (∼0.1°). In LabDCT for fully recrystallized samples, the minimum detectable grain size is of the order of >20–40 µm (Bachmann *et al.*, 2019 ▸). The spatial resolution for grains larger than 40 µm is shown to be ∼7 µm (McDonald *et al.*, 2015 ▸), while the most recent work reported a value of 4.4 µm (McDonald *et al.*, 2021 ▸). The spatial resolution is however unknown for grains of <40 µm. Although small-grain indexing might not be fully correct even with SR-DCT, the spatial resolution is much better than LabDCT. Therefore, the grain reconstruction from SR-DCT is considered to be the best possible ground truth to evaluate LabDCT reconstructions. To our knowledge, this is the first attempt to directly compare grain structures reconstructed by LabDCT with those obtained by SR-DCT. For a microstructure containing decorated GBs, Lab-DCT results have previously been compared with that obtained by phase-contrast tomography (King *et al.*, 2013 ▸). This method however only works for certain alloy systems, so here we have chosen to use the SR-DCT results as the ground truth.

We first show results for both the Laue focusing geometry and the magnified geometry, and we quantify differences in grain indexing and GSs with respect to the ground truth. Then, we unravel the causes of the differences by analyzing features of diffraction spots and comparing spots from experiments and forward simulations. Finally, we show how the number of theoretically expected and experimentally observed spots change in the magnified geometry compared with the Laue focusing geometry, and discuss parameters affecting the optimal setup for magnified geometries. All together the results are essential for advancing 3D characterization in home laboratories.

## 2. Experiments and data analysis   

### 2.1. Material and methods   

A fully recrystallized pure iron (99.99 wt% Fe) sample was chosen for the present investigation. A 50% cold-rolled pure iron plate was first annealed at 700°C for 30 min. Then, a cylindrical sample was cut with the axis along the rolling direction and electro-polished to a diameter of ∼500 µm. This sample was repeatedly characterized by SR-DCT (at room temperature) after additional annealing at 800°C for either ∼5 or 10 min. After 14 annealing steps and 15 SR-DCT measurements, the sample reached a state with many relatively large grains suitable for the present work. More details about the sample preparation and the SR-DCT measurements can be found in the work of Zhang *et al.* (2018 ▸).

LabDCT measurements were performed using a Zeiss Xradia 520 Versa X-ray microscope. A detector (2032 × 2032 pixels) with an effective pixel size of 3.36 µm was used. The scans were performed in a Laue focusing geometry, *i.e.* *L*_ss_ = *L*_sd_ = 13.0 mm, and a geometrically magnified condition with *L*_ss_ = 11.0 mm and *L*_sd_ = 18.0 mm, *i.e.* *L*_sd_/*L*_ss_ = 1.64. An exposure time of 500 s for each projection was adopted. A beamstop with an area of 2.5 × 2.5 mm was placed between the sample and the detector. A total of 181 diffraction projections were acquired during a full sample rotation of 360°. Both scans were comprised of recording diffraction projections followed by recording absorption tomographic projections, which were used for reconstructing the sample volume. An accelerating voltage of 160 kV and a power of 10 W were used for all scans. A total number of 1601 absorption tomographic projections were collected with an exposure time of 1.5 s per projection.

The grain reconstruction was performed with the commercial software *GrainMapper3D* version 2.2 developed by Xnovo Technology ApS (https://xnovotech.com/grainmapper3d-version-2-2/). The LabDCT projections were first processed by a rolling median correction through the image stack to remove most of the background noise, with the beamstop area excluded for further analysis. The diffraction spots were then segmented using a Laplacian of Gaussian based approach to create binary images, which subsequently were used for the grain reconstruction.

Spot segmentation is a crucial step for the quality of the grain reconstruction. In the present work, the segmentation parameters were optimized to render the best shape profile of the spots, based on visual inspection. More details about the spot segmentation are presented in Section S1 of the supporting information.

A fast geometric indexing algorithm (Bachmann *et al.*, 2019 ▸) was applied to index the orientation of each voxel within the gauge volume by performing forward simulations. Reconstruction parameters were kept the same for all the reconstructions. Completeness, defined as the fraction of observed and forward simulated signals expected in the binary images, is used to quantify the indexing confidence for each voxel. For both reconstructions, a minimum completeness was set at 45%, below which the indexing is rejected, and a maximum completeness was set at 85%, above which the indexing is taken for granted without any attempt for further improvement. Other reconstruction parameters, such as number of {*hkl*} families for indexing, finest degree of volume subdivision (termed maximum level in *GrainMapper3D*) and minimum misorientation (0.5° in this work) for merging voxels into the same grain, were kept the same for both reconstructions. Fitting of the detector position and tilt was employed to optimize the final reconstructed structures (Niverty *et al.*, 2019 ▸). The obtained grain structures were reconstructed with a voxel size of 2.5 µm. Notably, these reconstruction parameters are standard settings and do not necessarily yield the optimal reconstruction results for the magnified geometry. In spite of this, the parameters were kept constant, thereby avoiding effects of different reconstruction parameters on the grain-mapping results. The volumetric datasets are named as Lab-13-13 for the Laue focusing geometry and Lab-11-18 for the magnified geometry. More details about *GrainMapper3D* and its implementation for grain reconstruction can be found in the works of Bachmann *et al.* (2019 ▸) and Oddershede *et al.* (2019 ▸).

SR-DCT was performed on beamline ID11 at the European Synchrotron Radiation Facility, France, focusing on approximately the same gauge volume of the sample as carried out in the LabDCT scans. Details of the SR-DCT experiment were reported by Zhang *et al.* (2018 ▸). The grain structure was reconstructed with a voxel size of 1.5 µm. This dataset is named as SR-1.5.

To enable a direct comparison of the reconstructed grain structures between the SR-DCT and LabDCT datasets, the SR-1.5 dataset was rescaled to a voxel size of 2.5 µm and registered according to the LabDCT datasets. The details of the volume registration are given in Section S2. The resulting transformed dataset is named as SR-2.5, which is regarded as the ground truth in the following analysis.

### 2.2. Basis for grain comparison between the datasets   

First, the properties of all individual grains, including center of mass (COM), surface area, volume, size (taken as spherical equivalent diameter, *D*) and number of faces (taken as number of neighboring grains), were calculated. Second, the grains in the SR-2.5 dataset were paired to those in the two LabDCT datasets based on the grain orientation and position (see Section S2 for details of the pairing criteria). According to the pairing results, the grains are classified into four groups. The grains with a one-to-one match are referred to as ‘one-to-one indexed’. The grains in the SR-2.5 dataset where more than one match can be found in the LabDCT datasets are referred to as ‘one-to-multi indexed’. Grains found in the SR-2.5 dataset but not in the LabDCT datasets are referred to as ‘false-negatively indexed’, *i.e.* LabDCT fails to index those existing grains. Conversely, grains found in the LabDCT datasets but not in the SR-2.5 dataset are referred to as ‘false-positively indexed’, *i.e.* LabDCT wrongly indexes grains that actually do not exist.

For all good grain pairs (*i.e.* the one-to-one indexed grains), the GSs and GB deviations were quantified. The GS deviation of each voxel, ɛ_GS_, in each grain was calculated as the Euclidean distance between this voxel and the nearest voxel in the paired grain in the SR-2.5 dataset. ɛ_GS_ = 0 corresponds to a complete match, while ɛ_GS_ < 0 and ɛ_GS_ > 0 mean the voxel position in LabDCT is inside and outside a grain characterized by SR-DCT, respectively. A GS deviation map can thus be generated, visualizing shape differences. The average GS deviation, δ_GS_, for each grain in the LabDCT datasets was calculated by averaging the summation of absolute deviation values over all the non-zero voxels, *N*_voxel_(ɛ_GS_ ≠ 0),

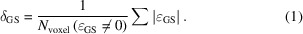

We also calculated the GB deviation, ɛ_GB_, for each voxel on the GBs. ɛ_GB_ was calculated as the Euclidean distance between a GB voxel in the SR-2.5 dataset and the nearest voxel in the paired grain in the LabDCT datasets. No sign was generated for ɛ_GB_. The average GB deviation, δ_GB_, for each grain in the LabDCT datasets was calculated by dividing the summation of ɛ_GB_ values by the total number of GB voxels in SR-2.5, *N*_voxel, GB_,

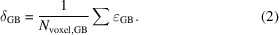


Fig. S3 of the supporting information illustrates a 2D scenario for computing maps of ɛ_GS_ and ɛ_GB_.

### 2.3. Analysis of diffraction spots   

With the reconstructed grains as input, we quantified spot features using a forward simulation model (Fang *et al.*, 2019 ▸, 2020 ▸), namely sizes, intensities and local background intensities around the spots (regions which are 20–30 pixels larger than the spot-bounding boxes excluding any other spots). For the four different groups of indexed grains, we performed further analysis on the comparison between the simulated and experimental spots.

## 3. Results   

### 3.1. Overall comparison   

#### 3.1.1. Reconstructed grain structures   

**Figure 1:**
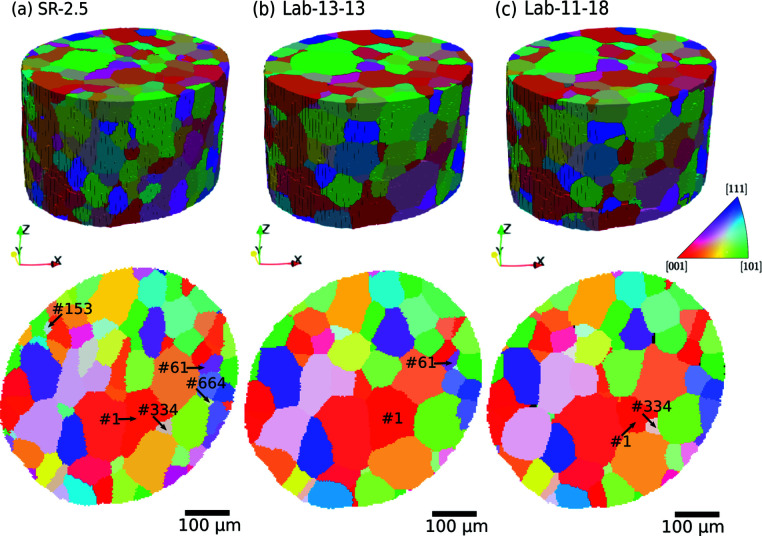
Grain structure datasets visualized in 3D in the top row and slices normal to the *Z* axis (sampled at a distance of 115 µm from the top surface) in the bottom row. (*a*) SR-2.5, (*b*) Lab-13-13 and (*c*) Lab-11-18 datasets. Some grains of interests are marked by numbers in the slices.

Fig. 1 ▸ shows the reconstructed 3D grain structures and 2D slices from the same position for the three datasets. The grains are colored throughout the article according to the inverse pole figure map along the *Z* direction shown in Fig. 1 ▸.

**Figure 2:**
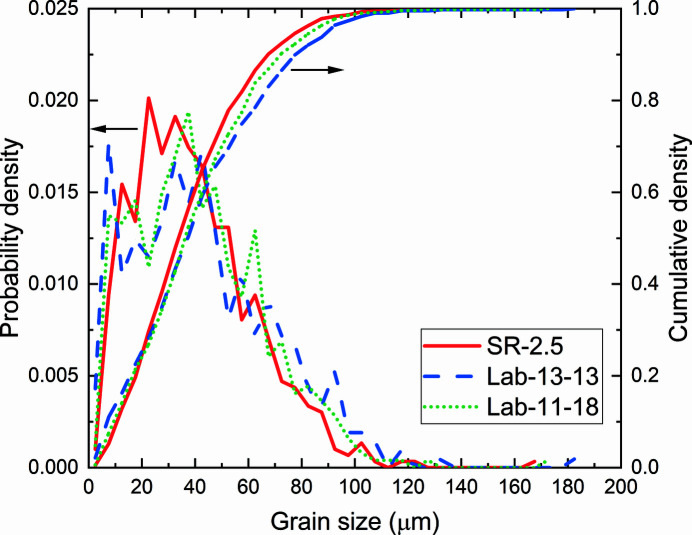
The grain size distribution and cumulative density for the three datasets.

The volumetric views in Fig. 1 ▸ show that both LabDCT results are qualitatively consistent with SR-2.5. The orientations and the approximate positions of most grains in both Lab-13-13 and Lab-11-18 agree with those in SR-2.5. As seen in Table 1 ▸, both Lab-13-13 and Lab-11-18 have fewer reconstructed grains than SR-2.5, leading to a slight overestimation of the average grain size. The differences are apparently more pronounced for Lab-13-13. Plots of grain size distributions for SR-2.5, Lab-13-13 and Lab-11-18 in Fig. 2 ▸ show that the cumulative densities of both Lab-13-13 and Lab-11-18 are largely consistent with that of SR-2.5, whilst main differences are seen for positions and values of local peaks in the probability density curves for the grain size distributions, especially for grains of <50 µm.

The differences in the number of reconstructed grains and the grain size distribution curves are related to differences in grain position, shape reconstruction and indexing. This can be observed by a careful look at the slices presented in Fig. 1 ▸. Some grains can be seen in the slice of SR-2.5, but not in the same slice of Lab-13-13 and/or Lab-11-18. For example, grain #61 can be seen both in the slices of SR-2.5 and Lab-13-13 but not in the slice of Lab-11-18. Grain #334 is present in the slices of SR-2.5 and Lab-11-18 but not in the slice of Lab-13-13. Similarly, grain #153 is seen in the slice of SR-2.5 but not in any of the two slices of the two LabDCT datasets. However, all these grains, which may not be seen in this particular slice, can actually be found in other slices, indicating that they are correctly indexed in the two LabDCT datasets but have different reconstructed positions and/or shapes compared with SR-2.5. Some other grains are false-negatively indexed in both LabDCT datasets. Such an example is grain #664, which is found in SR-2.5 but not in Lab-13-13 nor in Lab-11-18.

#### 3.1.2. Comparison of grain indexing   

**Figure 3:**
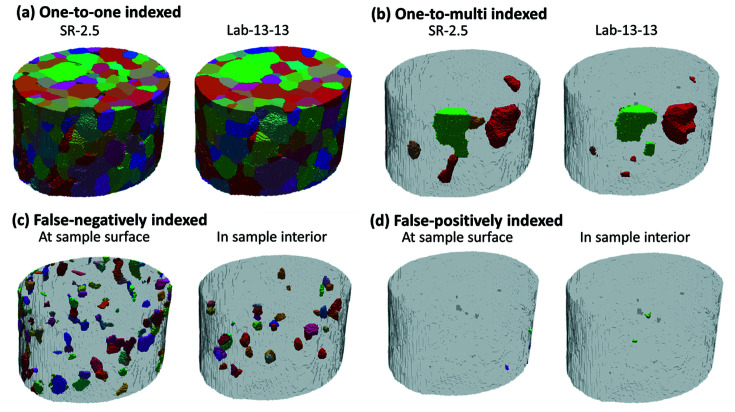
Visualization of the correspondence between indexed grains in the SR-2.5 and Lab-13-13 datasets. (*a*) One-to-one indexed grains, (*b*) one-to-multi indexed grains, (*c*) false-negatively indexed grains seen in the SR-2.5 dataset (but not in the Lab-13-13 dataset) that are at the sample surface or located in the sample interior and (*d*) false-positively indexed grains seen in the Lab-13-13 dataset that are at the sample surface or located in the sample interior.

All the grains separated into the aforementioned four indexing categories are visualized in Figs. 3 ▸ and 4 ▸. For the falsely indexed grains, we further show them separately for grains at the sample surface and in the sample interior. The key statistics are summarized in Table 2 ▸. More statistics on the falsely indexed grains can be found in Tables S2 and S3 of the supporting information.

A detailed analysis indicates that the majority of the grains, including the one-to-one and one-to-multi indexed grains, are true-positively indexed [see Figs. 3 ▸(*a*), 3 ▸(*b*), 4 ▸(*a*), 4 ▸(*b*) and Table 2 ▸]. The disorientation angles for all the true-positively indexed grain pairs are very small, on average Δθ = 0.02 ± 0.02° for both the Lab-13-13 and Lab-11-18 datasets (see Fig. S4 for more detail). There are 61 (∼15%) more true-positively indexed grains in Lab-11-18 than in Lab-13-13, which makes the mean grain size of Lab-11-18 closer to the ground-truth value (see Table 1 ▸). Most (>81%) of the false-negatively indexed grains are close to the sample surface and they are on average smaller than those in the sample interior [see Figs. 3 ▸(*c*), 4 ▸(*c*), Table 2 ▸ and Fig. S2]. Only a few (≤19) false-positively indexed small grains with an average size of ∼9.5 µm are distributed randomly in the bulk volumes [see Figs. 3 ▸(*d*), 4 ▸(*d*) and Table S3].

We selected three typical evaluation metrics, precision (*P*, in which false positives are penalized), sensitivity (*S*, also sometimes termed recall rate, in which false negatives are penalized) and *F*_1_ score (combining *P* and *S* into a single score; Chinchor, 1992 ▸), to quantitatively assess the performance of grain indexing in Lab-13-13 and Lab-11-18. The calculated results based on the number of true-positively, false-negatively and false-positively indexed grains are shown in Table 3 ▸. It is clearly seen that the *S* and *F*_1_ scores for Lab-11-18 are higher than those for Lab-13-13, while the precision values are very similar. This indicates that Lab-11-18 performs better in grain indexing than Lab-13-13. Notably, there are 13 grains smaller than 20 µm (with a minimum size of 7.6 µm) which are indexed successfully in Lab-11-18, whereas only three grains smaller than 20 µm (with a minimum size of 17.1 µm) are indexed in Lab-13-13. This indicates that the detection limit is improved when *L*_sd_/*L*_ss_ is increased from 1 to 1.64 for the present datasets.

### 3.2. Comparison for the one-to-one indexed grains   

All the 397 and 455 one-to-one indexed grains in the Lab-13-13 and Lab-11-18 datasets, respectively, are analyzed in detail with respect to their pairs in the SR-2.5 dataset, among which 388 grains are common for all three datasets. A comparison of average features including volume, surface area and number of faces for all the one-to-one indexed grains shows that the data in Lab-11-18 has smaller deviations from SR-2.5 compared with Lab-13-13 (see Fig. S5).

#### 3.2.1. Comparison of GSs and GB positions   

**Figure 5:**
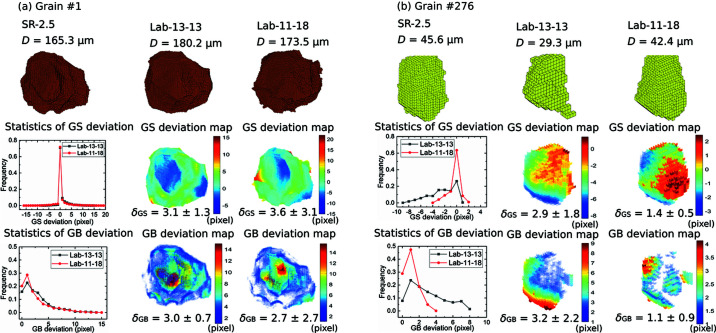
Comparison of GSs and GB positions between the two LabDCT datasets and SR-2.5 for (*a*) grain #1 and (*b*) grain #276. 3D voxelized volumes are shown in the top row. GS deviation statistics and maps are shown in the middle row and GB deviations are shown in the bottom row. The values of δ_GS_ and δ_GB_ are given for each deviation map. The grain size (denoted as *D*) is given for each reconstruction.

Two examples are shown in Fig. 5 ▸ comparing GSs and GB positions for a relatively large grain (grain #1) and a relatively small grain (grain #276) reconstructed in the LabDCT and SR-2.5 datasets. For the large grain #1, Fig. 5 ▸(*a*) shows that the distribution of the GS deviations between SR-2.5 and Lab-13-13 is quite similar to that between SR-2.5 and Lab-11-18, and for both cases more than 71% of the voxels are overlapping in the two datasets (*i.e.* >71% of the voxels have ɛ_GS_ = 0). The largest deviation in GS and GB position is found to be 15 pixels at the boundary of grain #1 for both LabDCT datasets, while a few voxels at the corner of the grain in the Lab-11-18 dataset are deviating even more. However, the grain size determined in Lab-11-18 is closer to that in SR-2.5 than Lab-13-13. This can be explained by the fact that there are 65% GB voxels with ɛ_GB_ ≤ 2 pixels in the Lab-11-18 dataset compared with 55% in the Lab-13-13 dataset.

For grain #276 shown in Fig. 5 ▸(*b*), the differences between the two LabDCT datasets in the deviation distribution curves are more pronounced. The deviations are attributed to differences in shapes and spatial shifts of the COM, both of which are smaller in Lab-11-18 than in Lab-13-13.

Comparing the average deviations (both δ_GS_ and δ_GB_), similar values are found for the large grain, whereas significantly smaller values are found for the small grain in the Lab-11-18 dataset compared with the Lab-13-13 dataset. This agrees well with the distribution curves of ɛ_GS_ and ɛ_GB_, indicating that δ_GS_ and δ_GB_ can be used to circumvent the need to examine the deviations for each voxel and serve as more concise metrics to characterize the quality of the shape reconstruction for each grain. It is also worth noting (see Fig. 5 ▸) that δ_GB_ is not equivalent to δ_GS_, although to some extent they are physically related. It is essential to compare both the GS and GB deviations for a comprehensive assessment of accuracies in the grain reconstruction.

**Figure 6:**
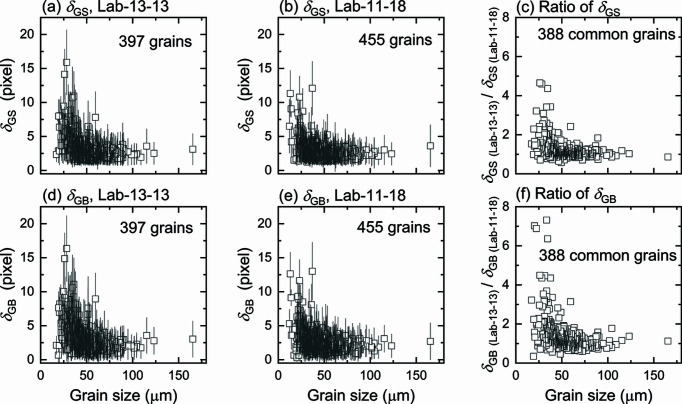
GS deviation δ_GS_ for (*a*) 397 grains in Lab-13-13 and (*b*) 455 grains in Lab-11-18, and GB deviation δ_GB_ for (*d*) Lab-13-13 and (*e*) Lab-11-18 as a function of grain size. (*c*) and (*f*) show the ratios of δ_GS_ and δ_GB_ for data in Lab-13-13 divided by that in Lab-11-18 for the 388 common one-to-one indexed grains. The size of the scatter bars in (*c*) and (*f*) are on average ∼1.7 but they are omitted in the plots for clarity.

To extend the comparison of δ_GS_ and δ_GB_, we plot them for each of all the one-to-one indexed grains as a function of grain size in Fig. 6 ▸. For both Lab-13-13 and Lab-11-18, the deviations fluctuate around small values (around one to three pixels) for the large grains, whereas they increase rapidly with decreasing grain size. The transition occurs at 20–45 µm. For some of the small grains, the deviations δ_GS_ and δ_GB_ are as large as the grain size or even larger. This is mainly due to both a significant difference in the determined grain size and dramatic spatial shifts of the grain COM positions (more details are shown in Figs. S6 and S7). Also the large deviations are more often found for the grains located at the sample surface and less frequently for grains in the sample interior. Interestingly, we found that the COM shifts of individual grains are strongly correlated with their distances to the center of the gauge volume (see Fig. S8). The longer the distance is, the larger the COM shift. This may be caused by the fact that the lengths of the beam at different projections change more for grains at the outer sample surface than for those near the center. This means that the attenuation of the beam, especially the low-energy part, varies more for the near-surface grains, resulting in more undetectable spots and thus a decrease in completeness for these grains. Since the calibration of the detector distance and tilt was performed for spots from grains with relatively high completeness values (this is a standard routine) during reconstruction, it leads to less accurate determination of both position and shape of near-surface grains. However, there might also be an intrinsic factor (related to the distribution of diffraction vectors) affecting the determination. This is however beyond the scope of the current work and needs further in-depth investigations.

Comparing the data between the two LabDCT datasets in Figs. 6 ▸(*a*) and 6 ▸(*b*), and 6 ▸(*d*) and 6 ▸(*e*), we see that both δ_GS_ and δ_GB_ in Lab-11-18 are generally smaller than Lab-13-13 for small grains, while their values for large grains are similar. It becomes even clearer when the ratios of δ_GS_ and δ_GB_ are plotted; see Figs. 6 ▸(*c*) and 6 ▸(*f*), respectively. The plots show that the ratios are ∼1.1 for large grains (*D* > 40 µm), whereas the ratios are higher for small grains (*D* ≤ 40 µm) and can be up to 7.3, indicating a significant improvement in spatial resolution for small grains in the Lab-11-18 dataset. Further statistics on δ_GB_ of large and small grains are plotted in Fig. S9.

Considering the ratios of δ_GB_ as an indicator of the spatial improvement in Lab-11-18 compared with Lab-13-13, we can conclude that for grains of <40 µm the spatial resolution is improved by a factor of 1.9 on average and can reach values as high as 7.3 when *L*_sd_/*L*_ss_ is increased from 1 to 1.64, whereas only a very small improvement with a factor of ∼1.2 is found for grains of >40 µm.

## 4. Discussion   

### 4.1. Reasons for differences in grain indexing   

The grain indexing results in differences of three types: one-to-multi, false-positively and false-negatively indexed (see Figs. 3 ▸, 4 ▸ and Table 2 ▸). To understand what causes the differences, we used forward simulations (Fang *et al.*, 2020 ▸) to compute the diffraction spots of each ‘problematic grain’ and compared those with the corresponding experimental spots in projections for rotation angles from −180 to 0° with a step size of 4°.

It was found that the one-to-multi indexing is only seen for grains with rather irregular shapes containing a narrow middle part, resulting in lower intensities within parts of the diffraction spots. Therefore, one single spot may be segmented as multiple spots, leading to multiple grains reconstructed in the LabDCT datasets. The one-to-multi indexing is however not considered to be a serious problem because it only fails to reconstruct a part of a grain while the indexing is still correct.

A few small grains (9.6 µm on average, see Table 2 ▸) are false-positively indexed. It was found that the simulated spots from these small grains are overlapped with other bigger spots, which are reflections from other larger grains. A similar observation was also reported by Lindkvist *et al.* (2021 ▸). In the current *GrainMapper3D*, the indexing of a voxel within the sample volume is accepted when the fraction of observed and forward simulated signals exceeds the preset minimum completeness (= 0.45 in our case). This algorithm can therefore not avoid the possibility that segmented spots in the binary images can be repeatedly used for indexing, resulting in false positives. Essentially, these false positives can be identified in a later version of *GrainMapper3D* by subtracting the overlapped frequencies to correct the completeness value, which would be below the minimum completeness and thus rejected for the indexing.

The majority of the differently indexed grains are false negatives (see Tables 2 ▸ and S1), which are indexed in SR-2.5 but not in the LabDCT datasets. These are all relatively small grains (<40 µm). They fall into three categories: category 1, indexed in neither of the two LabDCT datasets; category 2, indexed in Lab-11-18 but not in Lab-13-13; and category 3, indexed in Lab-13-13 but not in Lab-11-18.

Fig. S10 shows the number of grains falling into each of these categories for grains in the bulk and at the sample surface separately. Apparently most of the false negatives are located at the sample surface and are generally smaller than those in the sample interior (see Table S2). Since determination of COM positions for grains at the sample surface is systematically less precise than for the interior grains (according to Fig. S8), it is therefore possible that the chance to have false-negatively indexed grains at the sample surface becomes higher than in the sample interior. When we focus on the grains located in the sample interior, there are 16 grains belonging to category 1 and 19 grains belonging to category 2, whereas only one grain is found for category 3.

**Figure 7:**
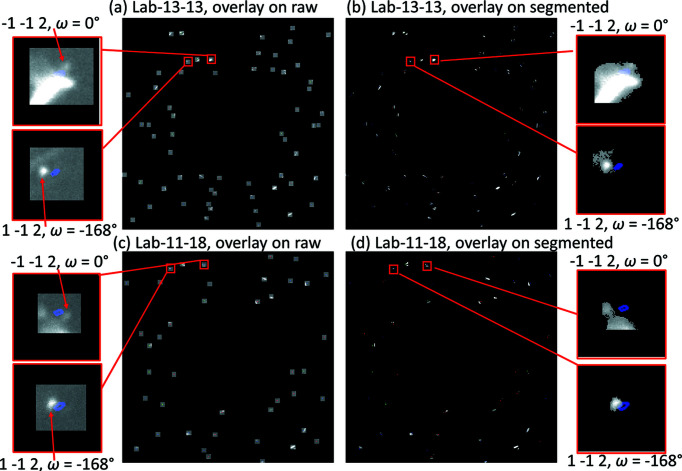
Overlay of forward simulated spots for grain #664 (a false-negatively indexed grain in both Lab-13-13 and Lab-11-18) onto experimental raw images and segmented images for (*a*), (*b*) Lab-13-13 and (*c*), (*d*) Lab-11-18. The forward simulation was performed from ω = −180 to 0° with a step of 4°. The resulting completeness for this grain is 0.37 and 0.21 for Lab-13-13 and Lab-11-18, respectively, which fall below the pre-set minimum completeness value (= 0.45). The rotation angle ω and (*hkl*) for each spot are given in the zoom-ins.

To understand the reason, we compared the simulated spots with the experimental spots for all these 36 interior false negatives. We can first confirm the existence of the false negatives indexed in SR-2.5 by the observation that the simulated spots (using the forward simulation tool; Fang *et al.*, 2020 ▸) are indeed strongly correlated with experimental spots in the raw images. An example is shown in Fig. 7 ▸ for grain #664 (*D* = 16.1 µm). By comparing the positions of simulated spots with those of observed [Figs. 7 ▸(*a*) and 7 ▸(*c*)] and segmented spots [Figs. 7 ▸(*b*) and 7 ▸(*d*)] for both geometries, reasons for false-negative indexing can be summarized as follows.

(*a*) Spatial shift. Although the spot is observable and well segmented, spatial shift leads to deviation from the theoretically expected position, *e.g.* spot 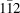 , ω = −168° for Lab-13-13. This is mainly due to sample drift and/or fluctuations of the data-acquisition system. Notably, Lab-11-18 is less sensitive to the spatial shift as it has a higher angular resolution than Lab-13-13.

(*b*) Under-segmentation. The experimental spot is too weak or connected to other stronger spots, and thus mis-segmented, *e.g.* spot 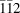 , ω = 0° for Lab-11-18. Notably, the spot ( 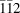 , ω = 0°) is much weaker than its pair ( 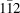 , ω = −168°). This is mainly due to a different spot energy, thus corresponding to a different photon flux as well as a different detective quantum efficiency of the detector system.

(*c*) Over-segmentation. The experimental spot is overlapped with other spots and does not segment to become separate from neighboring spots, *e.g.* spot 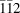 , ω = 0° for Lab-13-13.

(*d*) A low number of detectable spots due to the small grain size. This partly leads to a completeness value of 0.37 and 0.21 for Lab-13-13 and Lab-11-18, respectively, both of which are below the pre-set minimum completeness value, 0.45.

**Figure 8:**
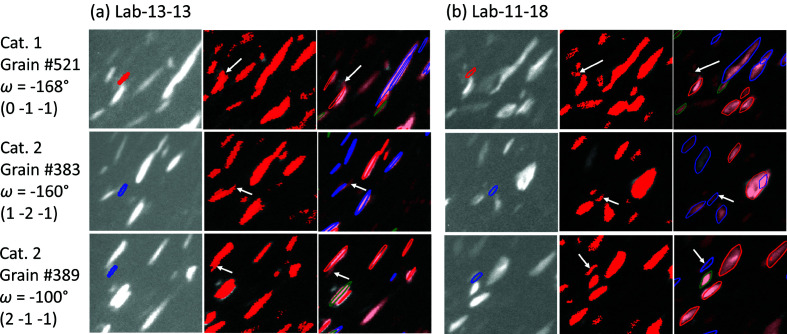
Overlay of the forward simulated spots (using the forward simulation tool; Fang *et al.*, 2020 ▸) for the false-negatively indexed grains of (*a*) Lab-13-13 and (*b*) Lab-11-18 onto the experimental image (left) together with the segmented image (middle) and the validation image (right) of the forward simulation from *GrainMapper3D*. Top-row images show spots for grain #521 (category 1), and middle and bottom rows show spots for grains #383 and #389 (category 2). The rotation angle ω and (*hkl*) for the spots are also given. Arrows indicate the spots that would be expected from these grains.

The effects of under- and over-segmentation leading to different indexing results from Lab-13-13 and Lab-11-18 are illustrated in Fig. 8 ▸. The figure shows overlays of simulated spots onto the experimental images, in parallel with a comparative view of segmented and validation images generated by *GrainMapper3D*. As an example, the 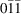 spot for grain #521 (*D* = 27.7 µm, mis-indexing in both LabDCT datasets) is over-segmented and thus connected to its neighbor in Lab-13-13, whereas this spot seems under-segmented but still connected to other spots in Lab-11-18, see Fig. 8 ▸.

As further shown for grains #383 (*D* = 37.1 µm) and #389 (*D* = 37.0 µm), the spots are segmented as connected to their neighbors in Lab-13-13, whereas they can be well segmented separately in Lab-11-18. Therefore, both grains are mis-indexed in Lab-13-13, while they are correctly indexed in Lab-11-18. Based on these observations, it is concluded that good indexing requires precise spot segmentation with a clear separation from neighboring spots and a small spot spatial shift.

Compared with Lab-13-13, the experimental images of Lab-11-18 better meet these demands. First, all the diffraction spots in each projection are more spread out in Lab-11-18 than Lab-13-13, meaning that the local background intensities for each spot are less influenced by neighboring spots. This is the most important difference and is especially beneficial for small and weak spots. We made an estimation on the average spot spacing λ_spot_ for each projection, calculated as λ_spot_ = [(*S*_det_ − *S*_spot_)/*N*_spot_]^1/2^, where *S*_det_ is the effective area of the detector recording diffraction signals (excluding the beamstop region), *S*_spot_ is the total area of the spots and *N*_spot_ is the number of spots on each projection. We found that λ_spot_ = 39 ± 1 pixels for Lab-13-13 and λ_spot_ = 58 ± 2 pixels for Lab-11-18 (here one pixel = 3.36 µm), confirming a broader spatial distribution of spots in Lab-11-18. It is worth noting that the effect of a decrease in number of spots (leading to an increase in λ_spot_) is significantly stronger than the counteractive effect of spot magnification (leading to a decrease in λ_spot_), thereby the actual λ_spot_ is increased for Lab-11-18. Second, Lab-11-18 has a higher angular resolution (corresponding to a smaller effective pixel size) and is thus less sensitive to fluctuations of sample drift and setup stabilities, resulting in less spot spatial shift. Third, the background noise in the radial direction is lower in Lab-11-18 than Lab-13-13. As shown in Fig. S11, the background noise ratio varies between 0.9 and 0.65 in the detector region where most of the spots are recorded, indicating a decrease of noise in Lab-11-18 compared with Lab-13-13. The decrease is largely due to two reasons: the same inelastic scatter is distributed over more pixels (thereby more uniform), and less intensity variation caused by high order {*hkl*} reflections is ‘diffused’ in the background for the magnified geometry compared with the Laue focusing geometry. A more detailed discussion of intensities and noise around individual spots is presented in Section 4.2.2.

The noise decrease for Lab-11-18 further enhances the chance of segmenting the small and weak spots as well as separating them from their neighbors. Although *Grain­Mapper3D* is able to deal with a certain degree of overlap for relatively large spots, as demonstrated by Bachmann *et al.* (2019 ▸), it becomes much harder when small spots that are partially overlapped/connected with others have to be segmented. This is the situation for the spots of most false-negatively indexed grains observed in the present investigation.

For the few grains belonging to category 3, no solid reason is found. We presume that it perhaps take place accidently or may be an effect of certain crystallographic orientations.

### 4.2. Improved spatial resolution for the common one-to-one indexed grains   

#### 4.2.1. Magnification of diffraction spots   

**Figure 9:**
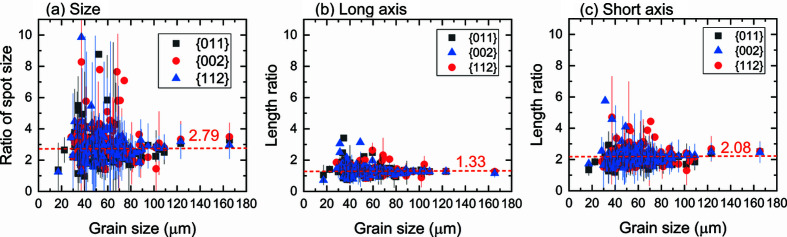
Ratios between the Lab-11-18 and Lab-13-13 datasets of (*a*) spot sizes and spot lengths of (*b*) long axis and (*c*) short axis as a function of grain size for the 130 common one-to-one indexed grains located in sample interior for the first three {*hkl*} planes.

Using the forward simulation model, we can track and trace spots for the same reflection of the same grain and thus compare the spot features at different *L*_sd_/*L*_ss_. We first concentrate on the changes in spot sizes, *i.e.* spot areas, with increasing *L*_sd_/*L*_ss_. Fig. 9 ▸(*a*) shows the ratios of spot sizes between *L*_sd_/*L*_ss_ = 1.64 (Lab-11-18) and *L*_sd_/*L*_ss_ = 1.00 (Lab-13-13). It can be seen that most of the spots are magnified and the size ratios for some spots vary significantly as indicated by the large scatter bars. Overall, the ratios appear to be independent of grain size and they also show little dependence on {*hkl*} families. We use the median value to represent the average, which is 2.79 for the spot-size ratio. Notably, the spot-magnification factor was considered when choosing the minimum spot size during segmentation (see Section S1).

To understand the effect of *L*_sd_/*L*_ss_ on the geometrical magnification of the spots better, we compared the ratios of lengths for the long and short axis of the spot separately [see Figs. 9 ▸(*b*) and 9 ▸(*c*), respectively]. In the Laue focusing geometry (*L*_sd_ = *L*_ss_, *e.g.* Lab-13-13) the diffraction occurring at different positions along the diffraction vector in the diffraction plane is focused to a point, while the diffracted beam from different positions perpendicular to the diffraction vector in the diffraction plane contributes to the length of the short axis. The resulting length of the spot short axis is related to the X-ray source size, Bragg angle, grain thickness in the diffraction plane and focusing distance in the Laue focusing geometry, as discussed by Kvardakov *et al.* (1997 ▸) and Stockmeier & Magerl (2008 ▸). However, in the magnified geometry (*L*_sd_ > *L*_ss_, *e.g.* Lab-11-18), the diffracted beam from different positions along the diffraction vector is no longer focused. Instead, it will also be magnified together with magnification of diffracted beam from different positions perpendicular to the diffraction vector in the diffraction plane, leading to variations in the ratios of the spot short axis ranging from ∼1 to 6 with a median average of 2.08 [see Fig. 9 ▸(*c*)]. The different magnifications of the long (perpendicular to the diffraction vector) and short axis (parallel to the projection of the diffraction vector on the detector) of the spot were also schematically illustrated and discussed by King *et al.* (2013 ▸).

The spot long axis is expected to be magnified by a factor of (*L*_sd_ + *L*_ss_)/2*L*_ss_ in the magnified geometry compared with the Laue focusing geometry and this magnification factor should be independent of grain dimensions. As seen in Fig. 9 ▸(*b*), the average magnified ratio for the long axes is found to be 1.33, which is in good agreement with the theoretical prediction: (11 + 18)/(2 × 11) = 1.32. Variations of the ratios can be seen but their magnitude is far smaller than that for the spot short axes. The combined contributions of the magnifications in the two directions led to the overall magnification factors with an average of 2.79, as shown in Fig. 9 ▸(*a*).

Although the magnification of the diffraction spots are nearly independent of grain size (see Fig. 9 ▸), the spot magnification does affect differently the accuracy in reconstructed GB position for different grain sizes. This is because the fraction of edge pixels of a spot (*i.e.* pixels at spot edge/total spot pixels, *f*_edge_) is roughly inversely proportional to the spot (or grain) size *D*, *i.e.* 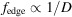 . The edge pixels are significantly influenced by the background noise. Therefore, a higher fraction of edge pixels leads to a poorer grain reconstruction and boundary position accuracy. With geometrical magnification, the fraction of edge pixels is reduced for all grains, resulting in improved 3D grain reconstruction (see Fig. 6 ▸). On top of that, for a given magnification factor for a magnified geometry (*L*_sd_/*L*_ss_ > 1) relative to the Laue focusing geometry (*L*_sd_/*L*_ss_ = 1), *m*, the reduction in fraction of edge pixels is inversely proportional to the grain size, as 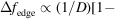 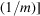 , where Δ*f*_edge_ = *f*_edge_(*L*_sd_/*L*_ss_ = 1) − *f*_edge_(*L*_sd_/*L*_ss_ ≥ 1). This explains why the improvement is more pronounced for smaller grains, as observed in Fig. 6 ▸.

Also, the angular resolution is improved when increasing *L*_sd_, thereby a better accuracy in determining the grain center was achieved. This improvement is particularly pronounced for small grains as their COM shifts compared with SR-2.5 are generally much larger than those for large grains (see Fig. S6).

#### 4.2.2. SNRs of diffraction spots   

**Figure 10:**
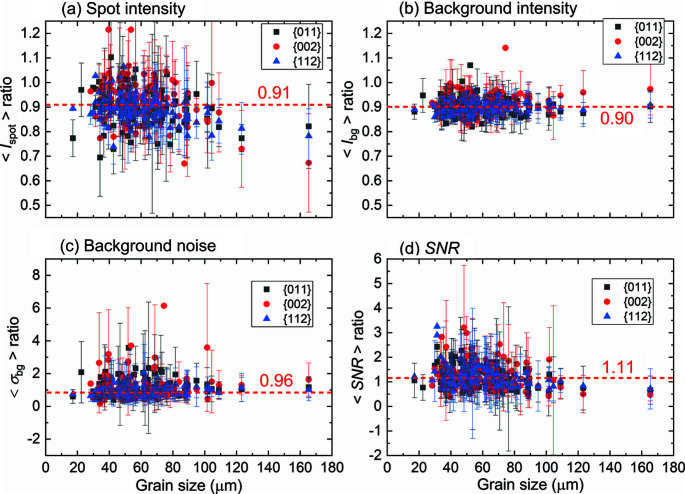
Ratios of (*a*) average spot intensities, (*b*) average local background intensities, (*c*) average local background noise and (*d*) SNRs from Lab-11-18 to Lab-13-13 datasets as a function of grain size. Dashed lines indicate the median values of all data points in each graph.

A sharp contrast between a spot and its local background noise is always beneficial for good spot segmentation, which is key for precise grain reconstruction. Using the forward simulation tool (Fang *et al.*, 2020 ▸), we determined the average spot intensity ( 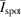 ), the local background intensity ( 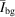 ) and the SNR for each spot. Here, SNR is defined as 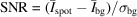 where σ_bg_ is the standard deviation of the local background noise distribution in the spot-bounding box excluding other spots. Figs. 10 ▸(*a*)–10 ▸(*d*) show ratios of 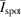 , 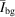 , σ_bg_ and SNR of Lab-11-18 to Lab-13-13. The figure shows that none of the ratios have a clear dependence on grain size. For ∼90% of the spots, 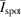 is lower in Lab-11-18 [the median of the ratios is 0.91, see Fig. 10 ▸(*a*)], which agrees with the expectation that the same integrated intensity diffracting from the same (*hkl*) of the same grain are now distributed over more pixels. Also, 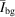 is lower in Lab-11-18 [the median ratio is 0.90, see Fig. 10 ▸(*b*)] for nearly all the spots (>99%), and for 55% of them σ_bg_ is lower in Lab-11-18 with a median ratio of 0.96 [see Fig. 10 ▸(*c*)]. The decrease in background intensity has a similar reason to the decrease of the spot intensity: the same amount of inelastic scattering is distributed over more pixels. All these changes of 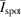 , 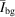 and σ_bg_ together lead on average to a slight increase in the SNR (median ratio of 1.11) for the same spot, while significant variations are also present, see Fig. 10 ▸(*d*).

Furthermore, the reduction in *L*_ss_ from 13 to 11 mm for the measurement of Lab-11-18 contributes to a reduction in the background noise, but this is not expected to significantly affect the observation and thus the grain reconstruction results. More details are discussed in Section S3.

To further substantiate the anticipation by excluding any subtle effect of *L*_ss_, we performed an additional LabDCT analysis using another iron sample with an average grain size of ∼24 µm in a focusing geometry (*L*_ss_ = *L*_sd_ = 11 mm) and a magnified geometry (*L*_ss_ = 11 mm and *L*_sd_ = 24 mm). Both measurements were performed in the same conditions and reconstructed with the same parameters. It was found that 691 grains are commonly indexed in both datasets (Lab-11-11 and Lab-11-24), while there are more than 180 grains in the sample interior which are indexed in Lab-11-24 but not in Lab-11-11. Although we do not have ground-truth data to directly validate the 180 extra indexed grains, we used our forward simulation model to compare the spots and found that most simulated spots from these grains were observed to perfectly match the experimental spots. This result further proves that the small change in *L*_ss_ from 13 to 11 mm in this study has only a minor impact on the obtained results.

The fact that the SNR does not get worse in Lab-11-18 [see Fig. 10 ▸(*d*)] and spots are more broadly spread (see Section 4.1) ensure that the diffraction spots (large or small) can be well segmented with better precision in defining the spot edges, resulting in improved spatial resolution.

### 4.3. Decrease of theoretical completeness by increasing *L*_sd_/*L*_ss_   

**Figure 11:**
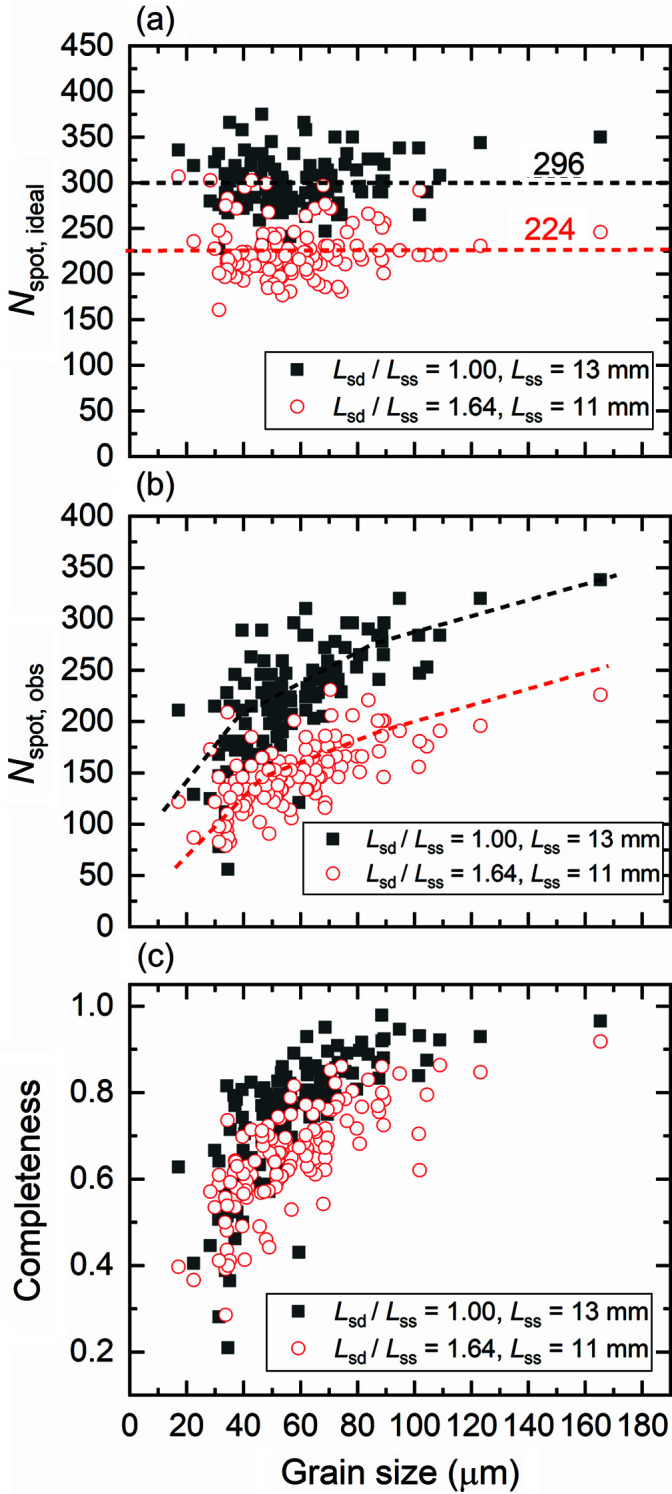
Number of (*a*) ideal (*N*_spot, ideal_) and (*b*) experimentally observed spots (*N*_spot, obs_) and (*c*) completeness at different *L*_sd_/*L*_ss_ for a total of 181 projections as a function of grain size obtained for the first three {*hkl*} families of the 130 common one-to-one indexed grains located in the sample interior. Dashed lines in (*a*) are average values and in (*b*) are guides to the eye.

Understanding the change of completeness is very important for optimizing the grain reconstructions when measuring with different geometries. Using the forward simulation tool (Fang *et al.*, 2020 ▸), we can compute the ideal number of spots (*N*_spot, ideal_) for different *L*_sd_/*L*_ss_ as a function of grain size [see Fig. 11 ▸(*a*)]. The experimentally observed number of spots (*N*_spot, obs_) is summarized in Fig. 11 ▸(*b*). As shown in Fig. 11 ▸(*a*), the theoretically expected number of spots is independent of grain size and decreases with increasing *L*_sd_/*L*_ss_. In contrast, the experimentally observed spot number depends strongly on grain size: the smaller the grain size, the fewer experimental spots are observable, and the decrease varies significantly for grains of <40 µm. The rate of decrease is slightly smaller in Lab-11-18 than Lab-13-13, suggesting that some diffraction spots from the small grains may have become indistinguishable in Lab-13-13 whereas they are still detectable in Lab-11-18. This is mainly due to a change of 2θ angles, which causes changes in spot energies and detective quantum efficiencies (see Fig. 10 in the work of Fang *et al.*, 2020 ▸), which in turn influence the detectability of the spots. More details are discussed in Section 4.4.

The completeness can be calculated by taking the ratio of *N*_spot, obs_ [Fig. 11 ▸(*b*)] to *N*_spot, ideal_ [Fig. 11 ▸(*a*)]. The results are shown in Fig. 11 ▸(*c*). The figure reveals a decrease in completeness with decreasing grain size. The completeness is determined by the detectability of the spot, which mainly depends on two factors: contrast, *i.e.* intensity above the background level 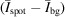 , and the local background noise σ_bg_. It is possible that the SNR increases, whereas the spot becomes undetectable because the contrast is too small while σ_bg_ is also small.

The fact that the completeness decreases with increasing *L*_sd_/*L*_ss_ indicates that the minimum completeness level may need to be set to a lower value for magnified geometries, especially when small grains have to be indexed. For example, when *L*_sd_/*L*_ss_ was further increased to 2.2 for the case of another sample with smaller grains (as shown in Section 4.3), we had to set a lower value of minimum completeness (35% instead of 45%) to ensure successful indexing of small grains. (Of course, in this case the same minimum completeness was used for the Laue focusing geometry to exclude the effects of different reconstruction parameters.) Whilst decreasing the minimum completeness value ensures more grains are indexed correctly, it also usually results in more false-positively indexed grains. Although false positives in principle can be identified and removed from the reconstruction with the help of a forward simulation model (*e.g.* Fang *et al.*, 2020 ▸), a suitable balance must be considered when setting the minimum completeness value during reconstruction.

### 4.4. Considerations concerning optimal magnified geometries   

Although the present experimental work was limited due to instrumental constraints imposed by the commercial setup (limited choices of detector, beamstop, source, *etc*., plus a limited range of distances between source, sample and detector), optimal data acquisition and geometry can be further explored with the aid of forward simulations. To support this further analysis, another set of measurements (*L*_sd_/*L*_ss_ = 1 and 2.2) using a different iron sample with smaller grains will be included here, even though no synchrotron ground-truth data are recorded for this sample. The measurements were also briefly discussed in Section 4.2.2.

With increasing *L*_sd_/*L*_ss_, the maximum accessible 2θ angle decreases { 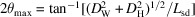 , where *D*_W_ and *D*_H_ are the detector width and height, respectively}, and the minimum also decreases [2θ_min_ = tan^−1^(*L*_beamstop_/2*L*_sd_), where *L*_beamstop_ is the side length of the beamstop]. Changes of both 2θ_max_ and 2θ_min_ inevitably lead to changes in the number of diffraction spots for different {*hkl*}s and spot energy ranges. The latter has a direct impact on the detectability of the spot since the detective quantum efficiency is sensitive to X-ray energies.

**Figure 12:**
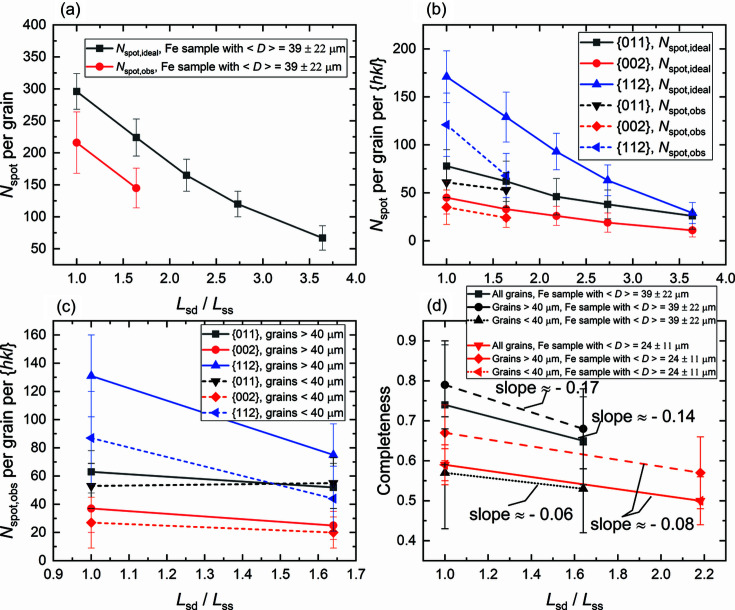
Average number of spots for the total of 181 projections and the completeness as a function of *L*_sd_/*L*_ss_. (*a*) *N*_spot, ideal_ and *N*_spot, obs_ per grain, (*b*) *N*_spot, ideal_ and *N*_spot, obs_ per grain for different {*hkl*}, and (*c*) *N*_spot, obs_ per grain for different {*hkl*} for grains larger or smaller than 40 µm for the iron sample with 〈*D*〉 = 39 ± 22 µm. (*d*) Average completeness for all the grains in the two iron samples (with 〈*D*〉 = 39 ± 22 µm and 〈*D*〉 = 24 ± 11 µm) subdivided into two categories: larger or smaller than 40 µm. All measurement conditions were kept constant; only *L*_sd_/*L*_ss_ was changed for any of the two samples.

We first analyze the change in the number of spots and completeness as a function of *L*_sd_/*L*_ss_ for the detector and beamstop size of our LabDCT setup. Fig. 12 ▸(*a*) shows that both the theoretical *N*_spot, ideal_ and the experimental *N*_spot, obs_ decrease with increasing *L*_sd_/*L*_ss_. Fig. 12 ▸(*b*) shows that *N*_spot_ decreases at different speeds for different {*hkl*}. The number of {112} spots decreases fastest both theoretically and experimentally. For *N*_spot, ideal_, it is because the spots from {112} are from the highest energies compared with the other two families and have the highest probability to hit outside the detector when increasing *L*_sd_/*L*_ss_. For *N*_spot, obs_, it is because only the higher-energy spots from {112} can be captured by the detector with increasing *L*_sd_/*L*_ss_, whereas the detective quantum efficiency decreases with increasing X-ray energy in the spot energy range for {112} (40.5–152.8 keV for *L*_sd_ = 18 mm).

Details of the distributions of spot energies for the first three {*hkl*} families are presented in Fig. S12. Notably, *N*_spot, obs_ for {011} decreases slowly, as shown in Fig. 12 ▸(*c*). *N*_spot, obs_ for {011} from grains smaller than 40 µm even increases for Lab-11-18 compared with Lab-13-13, while *N*_spot, obs_ for all the rest decreases. This can be understood by Fig. S12(*a*) showing that some spots from {011} in Lab-13-13 shift their energies from 22–33 keV to 33–45 keV in Lab-11-18, crossing the transition point where a partial increase (due to *K* edges of iodine and caesium, 33.17 and 35.98 keV, respectively) in the detective quantum efficiency is present for the CsI scintillation based detector (see Fig. 10 in Fang *et al.*, 2020 ▸). This shift is mainly caused by a slight decrease of *L*_ss_ from 13 to 11 mm and is thus important when comparing Lab-11-18 and Lab-13-13.

Fig. 12 ▸(*d*) shows that the completeness values decrease faster for larger grains than smaller grains in the sample with 〈*D*〉 = 39 ± 22 µm, while they decrease with a constant slope (= −0.08) in the sample with 〈*D*〉 = 24 ± 11 µm. This difference is mainly due to the unique behavior of *N*_spot, obs_ for {011}, as discussed above. Overall, the completeness is expected to decrease at a constant speed as a function of *L*_sd_/*L*_ss_ under the same measurement condition (primarily, the same exposure time). Since the sample with 〈*D*〉 = 24 ± 11 µm was measured with an exposure time of 300 s and a pixel binning of 2, the absolute completeness value for grains of <40 µm is higher than that in the other sample (measured with an exposure time of 500 s and no pixel binning).

As the total number of diffraction spots and completeness are both expected to decrease with increasing *L*_sd_/*L*_ss_, the following aspects must be considered when optimizing data acquisition and reconstruction for magnified geometries:

(i) *For optimal indexing, completeness values should not decrease too much*. Several measures can be taken to circumvent the decrease in completeness: (1) increasing the exposure time (increasing the actual exposure time and/or combining with pixel binning); (2) enhancing the detective quantum efficiencies, especially at high X-ray energies – this can be carried out by using either a high-energy sensitive detector or a low-resolution detector (usually with better detective efficiencies) placed at a large *L*_sd_/*L*_ss_; and (3) lowering the value for the minimum completeness during reconstruction but still keeping a balance not to generate too much false-positive indexing.

(ii) *For optimal shape reconstruction, the number of diffraction spots per grain should not be too few.* This can be realized by: (1) increasing the number of projections [see Fig. 12 ▸(*a*) for a guide to how many more projections is required for a given *L*_sd_/*L*_ss_], and (2) increasing the 2θ range with a proper combination of detector size and beamstop size.

Although it is not clear what is the minimum number of spots required for a good shape reconstruction for grains of different sizes, a previous study, dealing with optimizing the experimental conditions for the Laue focusing geometry, suggested that ∼30–40 projections (corresponding to ∼36–50 spots) result in reasonably good shape reconstruction for grain sizes of ∼75 µm (Lindkvist *et al.*, 2021 ▸). Analysis like that is needed to estimate how many spots are required for reconstructions of grains with sizes in different geometries.

## 5. Conclusions   

We have compared the 3D grain structures reconstructed from two LabDCT datasets, one measured in the usual Laue focusing geometry (*L*_sd_/*L*_ss_ = 1.00) and the other in a magnified geometry (*L*_sd_/*L*_ss_ = 1.64), with one dataset obtained by synchrotron DCT (considered as the ground truth). The physical origins of differences in grain indexing and shape reconstruction have been analyzed in detail. Additionally, parameters affecting the optimal setup at magnified geometries have been discussed. The following conclusions are drawn:

(*a*) Grain indexing can be significantly improved by increasing *L*_sd_/*L*_ss_. The grain detection limit was reduced from 17.1 to 7.6 µm when *L*_sd_/*L*_ss_ was increased from 1 to 1.64 for the sample studied in this work. Furthermore, many more small grains (<20 µm) are indexed successfully when *L*_sd_/*L*_ss_ is increased. The improved grain indexing in the magnified geometry is mainly due to a broader distribution of spots, a better angular resolution and a lower background noise in the LabDCT images, resulting in better spot segmentation with far less connection and overlap with neighboring spots.

(*b*) The spatial resolutions determined as accuracy in GB position for grains of <40 µm are improved by a factor of 1.9 on average and the factor can reach values as high as 7.3 when *L*_sd_/*L*_ss_ is increased from 1 to 1.64. The substantial improvement of GS reconstruction in the magnified geometry is mainly due to magnified diffraction spots, better determination of the grain COM positions and a more precise identification of spot edges.

(*c*) The completeness of individual grains decreases by ∼10% when *L*_sd_/*L*_ss_ is increased from 1 to 1.64. This decrease has to be considered when selecting optimal reconstruction parameters for measurements in magnified geometries.

This study has also shown that using forward simulation to trace experimental spots and comparing them with the simulated spots is essential in analyzing the underlying reasons for differences in grain reconstructions. Although the current work is limited to a case study of iron, the methodology for the data analysis is of a general nature and the conclusions are considered to be sample independent. In general, for an optimal LabDCT setup at magnified geometries, a combination of parameters including exposure time, detective quantum efficiency, detector size, beamstop size, number of projections and setting of the minimum completeness value should be tuned to maximize the number of detectable spots.

## 6. Related literature   

The following references are cited in the supporting information for this article: Lind (2013 ▸).

## Supplementary Material

Supporting information including supporting tables and figures. DOI: 10.1107/S2052252521003730/fc5052sup1.pdf

## Figures and Tables

**Figure 4:**
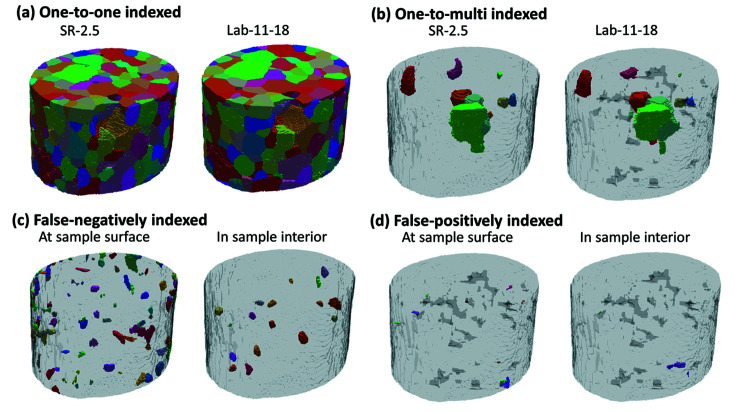
Visualization of the correspondence between indexed grains in the SR-2.5 and Lab-11-18 datasets. (*a*) One-to-one indexed grains, (*b*) one-to-multi indexed grains, (*c*) false-negatively indexed grains seen in SR-2.5 dataset (but not in the Lab-11-18 dataset) that are at the sample surface or located in the sample interior and (*d*) false-positively indexed grains seen in the Lab-11-18 dataset that are at the sample surface or located in the sample interior.

**Table 1:** Grain parameters obtained by the three methods The grain size reported is expressed by the mean value and standard deviation.

Dataset name	Number of grains	Grain size (µm)
SR-2.5	596	39.6 ± 22.4
Lab-13-13	418	43.1 ± 26.9
Lab-11-18	495	41.8 ± 24.3

**Table 2:** Statistics of the grains within the four different indexing categories in the LabDCT datasets *N* denotes the number of grains, 〈*D*〉 is the mean grain size, σ_*D*_ is the standard deviation of the grain size distribution and *f*_V_ is the volume fraction. The statistics of one-to-one and one-to-multi indexed grains are merged and labeled as true-positively indexed. The values in the bracket of *N* for the true positives are the number of one-to-one indexed grains.

LabDCT dataset	True-positively indexed				False-negatively indexed				False-positively indexed			
	*N*	〈*D*〉 (µm)	σ_*D*_ (µm)	*f*_V_	*N*	〈*D*〉 (µm)	σ_*D*_ (µm)	*f*_V_	*N*	〈*D*〉 (µm)	σ_*D*_ (µm)	*f*_V_
Lab-13-13	404 (397)	49.9	19.7	0.977	192	18.1	7.6	0.022	7	9.6	3.8	0.0001
Lab-11-18	465 (455)	46.6	20.3	0.992	131	14.9	5.9	0.008	19	9.5	4.0	0.0003

**Table 3:** Performance of grain indexing TP, FP and FN denote true positive, false positive and false negative, respectively. *P* and *S* refer to precision and sensitivity, respectively.

LabDCT dataset	*P* = TP/(TP + FP)	*S* = TP/(TP + FN)	*F*_1_ = 2*P* *S*/(*P* + *S*)
Lab-13-13	0.983	0.678	0.802
Lab-11-18	0.961	0.780	0.861

## References

[bb1] Bachmann, F., Bale, H., Gueninchault, N., Holzner, C. & Lauridsen, E. M. (2019). *J. Appl. Cryst.* **52**, 643–651. 10.1107/S1600576719005442 PMC6557177 31236094

[bb2] Chinchor, N. (1992). *Proceedings of the 4th Conference on Message Understanding*, pp. 22–29. San Mateo: Morgan Kaufmann.

[bb3] Fang, H., Jensen, D. J. & Zhang, Y. (2019). *IOP Conf. Series: Mater. Sci. Eng.* **580**, 012030.

[bb4] Fang, H., Juul Jensen, D. & Zhang, Y. (2020). *Acta Cryst.* A**76**, 652–663. 10.1107/S2053273320010852 PMC7598096 33125349

[bb5] Hayashi, Y., Hirose, Y. & Seno, Y. (2015). *J. Appl. Cryst.* **48**, 1094–1101.

[bb6] Henningsson, N. A., Hall, S. A., Wright, J. P. & Hektor, J. (2020). *J. Appl. Cryst.* **53**, 314–325. 10.1107/S1600576720001016 PMC7133059 32280319

[bb7] Jakobsen, A. C., Simons, H., Ludwig, W., Yildirim, C., Leemreize, H., Porz, L., Detlefs, C. & Poulsen, H. F. (2019). *J. Appl. Cryst.* **52**, 122–132.

[bb8] Johnson, G., King, A., Honnicke, M. G., Marrow, J. & Ludwig, W. (2008). *J. Appl. Cryst.* **41**, 310–318.

[bb9] Juul Jensen, D. & Zhang, Y. B. (2020). *Curr. Opin. Solid State Mater. Sci.* **24**, 100821.

[bb10] King, A., Reischig, P., Adrien, J. & Ludwig, W. (2013). *J. Appl. Cryst.* **46**, 1734–1740.

[bb11] King, A., Reischig, P., Adrien, J., Peetermans, S. & Ludwig, W. (2014). *Mater. Charact.* **97**, 1–10.

[bb12] Kvardakov, V. V., Somenkov, V. A., Lynn, J. W., Mildner, D. F. R. & Chen, H. (1997). *Physica B*, **241**, 1210–1212.

[bb13] Larson, B. C., Yang, W., Ice, G. E., Budai, J. D. & Tischler, J. Z. (2002). *Nature*, **415**, 887–890. 10.1038/415887a 11859363

[bb14] Lei, X. C., Zhang, Y. B., Sun, J., Bachmann, F., Yang, X. F., Sanders, R. E. & Juul Jensen, D. (2021). *Mater. Res. Lett.* **9**, 65–70.

[bb38] Lind, J. F. (2013). PhD thesis, Carnegie Mellon University, Pittsburgh, Pennsylvania, USA.

[bb15] Lindkvist, A., Fang, H., Juul Jensen, D. & Zhang, Y. (2021). *J. Appl. Cryst.* **54**, 99–110. 10.1107/S1600576720014673 PMC7941312 33833643

[bb16] Ludwig, W., Schmidt, S., Lauridsen, E. M. & Poulsen, H. F. (2008). *J. Appl. Cryst.* **41**, 302–309.

[bb17] McDonald, S. A., Burnett, T. L., Donoghue, J., Gueninchault, N., Bale, H., Holzner, C., Lauridsen, E. M. & Withers, P. J. (2021). *Mater. Charact.* **172**, 110814.

[bb18] McDonald, S. A., Holzner, C., Lauridsen, E. M., Reischig, P., Merkle, A. & Withers, P. J. (2017). *Sci. Rep.* **7**, 5251. 10.1038/s41598-017-04742-1 PMC5507940 28701768

[bb19] McDonald, S. A., Reischig, P., Holzner, C., Lauridsen, E. M., Withers, P. J., Merkle, A. P. & Feser, M. (2015). *Sci. Rep.* **5**, 14665. 10.1038/srep14665 PMC4615976 26494523

[bb20] Niverty, S., Sun, J., Williams, J., Bachmann, F., Gueninchault, N., Lauridsen, E. M. & Chawla, N. (2019). *JOM*, **71**, 2695–2704.

[bb21] Oddershede, J., Sun, J., Gueninchault, N., Bachmann, F., Bale, H., Holzner, C. & Lauridsen, E. M. (2019). *Integr. Mater. Manuf. Innov.* **8**, 217–225.

[bb22] Pankhurst, M. J., Gueninchault, N., Andrew, M. & Hill, E. (2019). *MinMag*, **83**, 705–711.

[bb23] Poulsen, H. F. (2004). *Three-Dimensional X-ray Diffraction Microscopy: Mapping Polycrystals and Their Dynamics*. Berlin: Springer.

[bb24] Poulsen, H. F. (2012). *J. Appl. Cryst.* **45**, 1084–1097.

[bb25] Poulsen, H. F. (2020). *Curr. Opin. Solid State Mater. Sci.* **24**, 100820.

[bb26] Poulsen, H. F., Nielsen, S. F., Lauridsen, E. M., Schmidt, S., Suter, R. M., Lienert, U., Margulies, L., Lorentzen, T. & Juul Jensen, D. (2001). *J. Appl. Cryst.* **34**, 751–756.

[bb27] Reischig, P., King, A., Nervo, L., Viganó, N., Guilhem, Y., Palenstijn, W. J., Batenburg, K. J., Preuss, M. & Ludwig, W. (2013). *J. Appl. Cryst.* **46**, 297–311.

[bb28] Reischig, P. & Ludwig, W. (2020). *Curr. Opin. Solid State Mater. Sci.* **24**, 100851.

[bb29] Renversade, L., Quey, R., Ludwig, W., Menasche, D., Maddali, S., Suter, R. M. & Borbély, A. (2016). *IUCrJ*, **3**, 32–42. 10.1107/S2052252515019995 PMC4704077 26870379

[bb30] Simons, H., King, A., Ludwig, W., Detlefs, C., Pantleon, W., Schmidt, S., Stöhr, F., Snigireva, I., Snigirev, A. & Poulsen, H. F. (2015). *Nat. Commun.* **6**, 6098. 10.1038/ncomms7098 PMC4354092 25586429

[bb31] Stockmeier, M. & Magerl, A. (2008). *J. Appl. Cryst.* **41**, 754–760.

[bb32] Sun, J., Holzner, C., Bale, H., Tomita, M., Gueninchault, N., Bachmann, F., Lauridsen, E. M., Inaguma, T. & Kimura, M. (2020). *ISIJ Int.* **60**, 528–533.

[bb33] Sun, J., Yu, T., Xu, C., Ludwig, W. & Zhang, Y. (2018). *Scr. Mater.* **157**, 72–75.

[bb34] Sun, J., Zhang, Y., Lyckegaard, A., Bachmann, F., Lauridsen, E. M. & Juul Jensen, D. (2019). *Scr. Mater.* **163**, 77–81.

[bb35] Suter, R. M., Hennessy, D., Xiao, C. & Lienert, U. (2006). *Rev. Sci. Instrum.* **77**, 123905.

[bb36] Syha, M., Trenkle, A., Lödermann, B., Graff, A., Ludwig, W., Weygand, D. & Gumbsch, P. (2013). *J. Appl. Cryst.* **46**, 1145–1150. 10.1107/S002188981301580X PMC3769074 24046507

[bb37] Zhang, J., Zhang, Y., Ludwig, W., Rowenhorst, D., Voorhees, P. W. & Poulsen, H. P. (2018). *Acta Mater.* **156**, 76–85.

