# Supplementary material for: Improved grain mapping by laboratory X-ray diffraction contrast tomography
Source: IUCrJ. 2021 May 7;8(Pt 4):559–73. doi: 10.1107/S2052252521003730 (PMC8256707; doi:10.1107/S2052252521003730)
Supplement: Supplementary file 1 [file m-08-00559-sup1.pdf]

IUCrJ

Volume 8 (2021)

Supporting information for article:

Improved grain mapping by laboratory X-ray diffraction contrast tomography

H. Fang, D. Juul Jensen and Y. Zhang

S1. Spot segmentation in GrainMapper3D

Binarized diffraction spots from the rolling median corrected diffraction images can be obtained in several ways in GrainMapper3D. The Laplacian of Gaussian (LoG) based spot segmentation has shown to be one of the most effective ways. In the LoG approach, a background value is subtracted from each input image, which subsequently is smoothed with a Gaussian filter, and then convoluted with a Laplacian filter, resulting in a LoG image. Diffraction spots are subsequently segmented by applying a threshold value, which is defined as a percentage of the maximum intensity of each connected component in every LoG image. The detailed procedure is illustrated in Algorithm 1 and can also be found in (Lind 2013). Since very small spots are most likely just noise, only the spots containing more than a certain number of pixels (defined as *Min Spot Size*) are selected. In the present work, we used this LoG approach to segment the spots in each diffraction image. Due to the geometrical magnification, some bright spots in the diffraction images of Lab-11-18 become 'fatter' and their shapes cannot be segmented with the LoG approach alone although it worked very well for images of Lab-13-13. Therefore, we combined the LoG approach with a simple thresholding, in which a single threshold and a minimum spot size were applied to segmented spots thereby optimizing the segmentation quality for Lab-11-18.

The segmentation parameters for the two datasets are listed in Table S1. It should be pointed out that the *Min Spot Size* in the LoG method for Lab-11-18 was set as 15 pixels, which is 3 times that for Lab-13-13, in agreement with the magnification factor of the diffraction spot (which is about 2.79, see Figure 9a). This setting means that an unbiased segmentation for the two LabDCT datasets is obtained. Figure S1 shows an example of the diffraction projections and the corresponding binary images.

Algorithm 1. Laplacian of Gaussian based spot segmentation

Require: input image *Im*, standard deviation σ, threshold percentage *Percent*, background value *Bkg*.

1) $Im_{\text{sub}} = Im - Bkg$;

2) Im_{Gaussian} = GaussianImageFilter(Im_{sub}, σ);

3) $Im_{\text{Laplacian}}$ = LaplacianImageFilter(Im_{Gaussian});

4) $Im_{\text{LoG}} = -\, Im_{\text{Laplacian}}$;

5) $bIm = Im_{\text{LoG}} > 0$;

6) *vListClusters* = ConnectedComp(*bIm*);

7) *vAcceptedPixels* = [];

8) **for** $j = 1$; $j \leq nNumberOfClusters$; j + + **do**

9)   *nThreshVal* = *Percent* * max(*vListClusters*[j]. Pixel[:]);

10)   **for** $k = 1$; $k \leq$ *vListClusters*[j]. Pixel. length(); k + + **do**

        if *vListClusters*[j]. Pixel. Intensity > *nThreshVal* **then**

            vAcceptPixels. Add(*vListClusters*[j]. Pixel[k]);

        end if

11)   **end for**

12) **end for**

Table S1 Parameters for spot segmentation**.**

Segmentation method	Parameters	Lab-13-13	Lab-11-18
LoG	Background value, *Bkg*	2	1
	Standard deviation, σ (pixel)	10	10
	Percent	5%	15%
	Min Spot Size (pixel)	5	15
Single thresholding	Background threshold	-	5
	Min Spot Size (pixel)	-	3

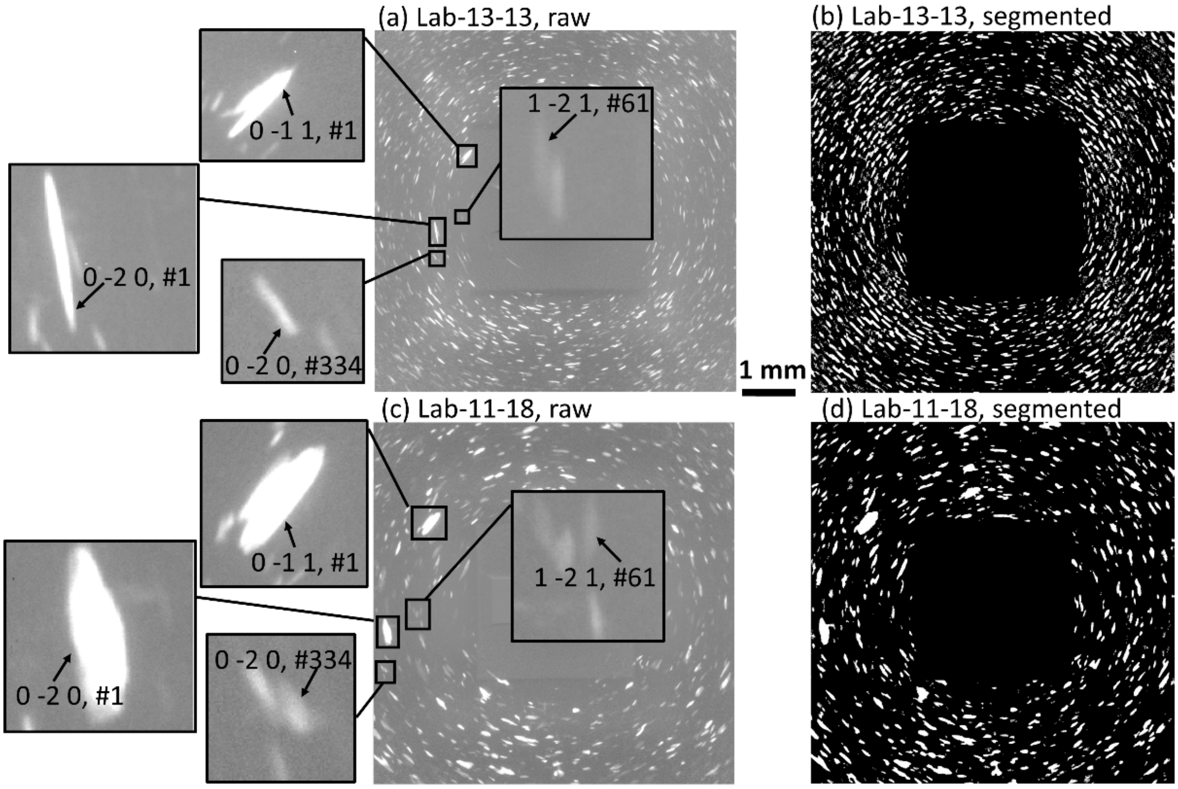

Figure S1 Experimental LabDCT projections of (a) Lab-13-13 and (c) Lab-11-18 at a rotation angle of 36° and the corresponding spot segmented images (binary images) using GrainMapper3D shown in (b) and (d), respectively. Zoom-ins show the common *hkl* diffraction spots of grain #1, #61 and #334 (all are marked in Figure 1). The scale bar is common to all the images excluding the zoom-ins. Both (a) and (c) are shown in the same grey value range (0 – 175).

S2. Volume registration

The volume registration consists of two processes: scaling SR-1.5 to the same voxel size of 2.5 μm as Lab-13-13 and rotating and shifting the grain volume after scaling to maximize the correspondence between the final transformed dataset and the reference dataset Lab-13-13. The whole process for each position (represented as a voxel *i*, defined in *x*, *y* and *z* coordinates with units of μm) in the raw dataset Sync-1.5 $\boldsymbol{A}_0{}^i$ can be expressed as:

$$\boldsymbol{A}^i = \boldsymbol{R}\boldsymbol{S}\boldsymbol{A}_0^i + \boldsymbol{T}, \tag{S1}$$

where $\boldsymbol{A}^i$ is the corresponding position in the new dataset after volume transformation, $\boldsymbol{S}$ is a 3×3 matrix defining scaling and any distortion, $\boldsymbol{R}$ is a 3×3 matrix representing rotation and $\boldsymbol{T}$ is a 3×1 matrix representing translation. With the sample distortion expected to be negligible, $\boldsymbol{S}$ can be written as:

$$\boldsymbol{S} = \begin{pmatrix} m & 0 & 0 \\ 0 & m & 0 \\ 0 & 0 & m \end{pmatrix}, \quad \text{(S2)}$$

where m = 1.67 defines the magnification factor for scaling the voxel size from 1.5 to 2.5 □m.

To find the optimal ***R*** and ***T,*** yielding the best match between the SR dataset after transformation and Lab-13-13, we determined ***R*** and ***T*** separately. We derived the values of ***R*** by minimizing an objective function that accounts for the disorientation angle (□□) of each grain pair j. This objective function □$_R$ can be expressed as:

$$\Delta_{\boldsymbol{R}} = \frac{1}{N_{pair}} \sum_{j}^{N_{pair}} \Delta\theta_j, \quad \text{(S3)}$$

N_{pair} is the total number of grain pairs (we will discuss how to determine the grain pairs later). By visually inspecting the positions and orientations of grains in SR-1.5 and Lab-13-13, we initially confirmed 12 grain pairs (thus, $N_{pair,\ initial}$ = 12), which were used as input to minimize Equation S3 and obtain the first guess of ***R***. To find all possible grain pairs, each grain in SR-1.5 (i_{SR}) was compared to any one of the grains in Lab-13-13 (i_{Lab}). The two grains are considered as a pair when the following two constraints are fulfilled:

$$\Delta\theta(i_{SR}, i_{Lab}) \leq threshold\ 1, \quad \text{(S4)}$$

$$\frac{\sqrt{\sum_{k}^{N_{pair,initial}} \left(d(i_{SR}, k_{SR}) - d(i_{Lab}, k_{Lab})\right)^2}}{N_{pair,initial}} \leq threshold\ 2, \quad \text{(S5)}$$

where $N_{pair,\ initial}$ is the number of grain pairs initially confirmed, $d(i_{SR}, k_{SR})$ and $d(i_{lab}, k_{lab})$ are Euclidean distances between the centroid of grain i and the centroid of grain k among the initially confirmed grains in the SR-1.5 and Lab-13-13 datasets, respectively. After the iterative check and comparison, we set the threshold values as *threshold 1* = 0.25° and *threshold 2* = 1.2D where D is the grain size. With these values, 397 grain pairs were uniquely identified between SR-1.5 and Lab-13-13. These grains pairs were then used for minimizing Equation S3 to derive the final values of ***R***.

To determine the values of ***T***, the second objective function □$_T$ was minimized:

$$\Delta_{\boldsymbol{T}} = \frac{1}{N_{pair}} \sum_{j}^{N_{pair}} d(j_{SR}, j_{Lab}), \quad \text{(S6)}$$

where N_{pair} is the number of grain pairs (in this case N_{pair} = 397) and $d(j_{SR}, j_{Lab})$ is the Euclidean distance between the centroid of the grain j in the dataset after SR-1.5 was scaled and rotated, and the centroid of the corresponding grain j in Lab-13-13.

Since the probed sample volumes were not exactly the same across the three datasets, each of the three volumes was cropped in all the three directions to obtain the same common sample volume. The final transformed dataset of SR-1.5 is named as SR-2.5.

Comparing SR-2.5 to SR-1.5, the average grain size is increased by about 0.3 μm and only three grains smaller than 4 μm are left out by the transformation. Additionally, very small differences in grain

shape and boundary positions can be observed as shown in Figure S2. Therefore, the data transformation is considered to have negligible effect on the fidelity of the grain structure.

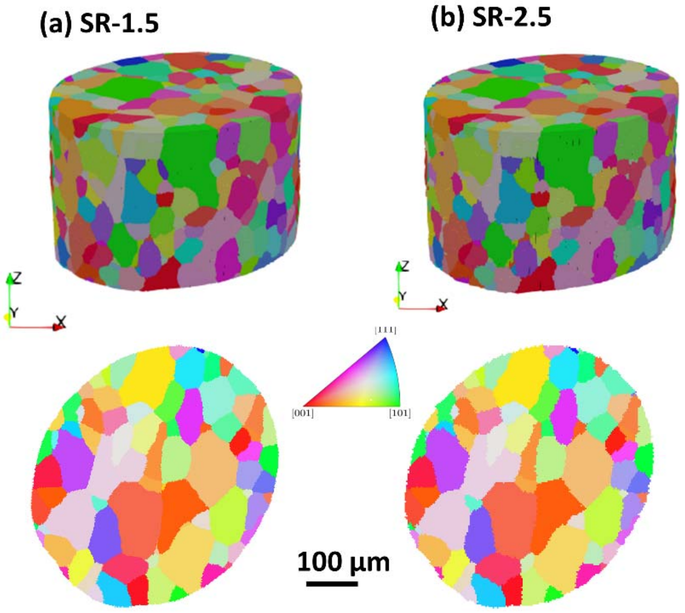

Figure S2 The grain structure visualized in 3D in the top row and slices normal to the *Z* axis (sampled at a distance of 115 µm from the top surface) in the bottom row for datasets (a) SR-1.5 and (b) SR-2.5.

S3. Analysis of effects of decreasing L_{ss} on the reduction in noise for a constant L_{sd}

As LabDCT adopts a conical X-ray beam, the cone angle (α) increases when L_{ss} decreases from 13 mm to 11 mm, while keeping L_{sd} = 18 mm. Here, the cone angle is calculated by $\alpha = atan\left(\frac{D_{det}}{2(L_{ss}+L_{sd})}\right)$ (where D_{det} is the width of the detector, D_{det} = 6.83 mm in this work). The resulting solid angle (Ω) seen by the same detector thus increases (because $\Omega = 2\pi(1 - cos(\alpha))$), which leads to an increase of photon flux (φ) for L_{ss} = 11 mm compared to L_{ss} = 13 mm. As the background noise, which can be viewed as relative counting error (σ_{bg}) in the present work, is inversely proportional to square root of photon flux and thus the solid angle, *i.e.* $\sigma_{bg} \propto \sqrt{1/\varphi} \propto \sqrt{1/\Omega}$, the decrease of L_{ss} from 13 to 11 mm leads to a decrease of background noise. Assuming the noise is only due to inelastic scattering from the sample while ignoring other less significant noise origins such as the aperture, we can now calculate values of α and thus derive the ratio of σ_{bg} for L_{ss} = 13 mm to L_{ss} = 11 mm with a constant L_{sd} (= 18 mm).

Calculation shows that the σ_{bg} ratio is 1.07, indicating a 7% decrease in noise by decreasing L_{ss} from 13 to 11 mm. This 7% decrease in noise is less significant than the 11% increase of the *SNR* median

ratio (see Figure 10d). Therefore, it is anticipated that a slight decrease of L_{ss} (from 13 to 11 mm) would not significantly affect the results in this work.

Other supplementary figures:

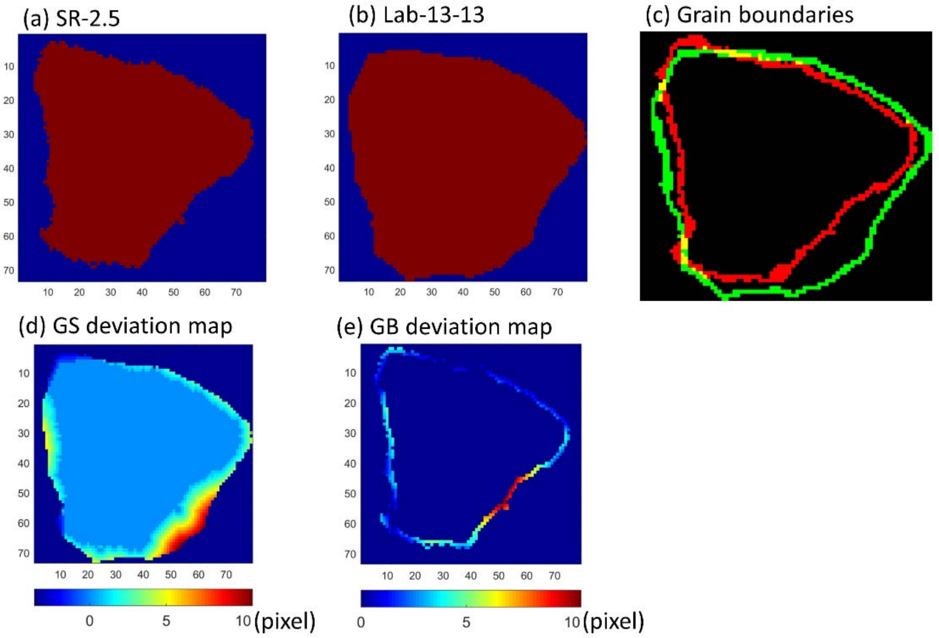

Figure S3 Example extracted from grain #1 showing the calculation of the grain shape deviation (ε_{GS}) and grain boundary deviation (ε_{GB}). A slice of grain #1 in (a) SR-2.5 and (b) Lab-13-13; (c) identified grain boundaries where red is for SR-2.5 and green is for Lab-13-13; (d) grain shape (GS) deviation map and (e) grain boundary (GB) deviation map. We shall point out that this 2D example is shown for easy visualization. Our actual calculation and the results reported in this paper are in 3D.

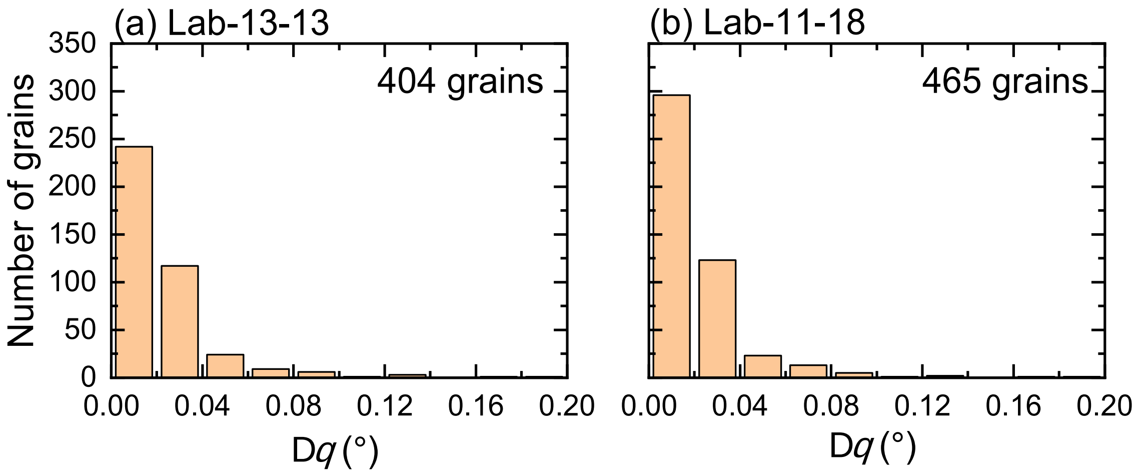

Figure S4 Distribution of disorientation angle $\Delta\theta$ for the true-positively indexed grains between the two LabDCT datasets and the SR-2.5 dataset. There are in total (a) 404 grains in Lab-13-13 and (b) 465 grains Lab-11-18.

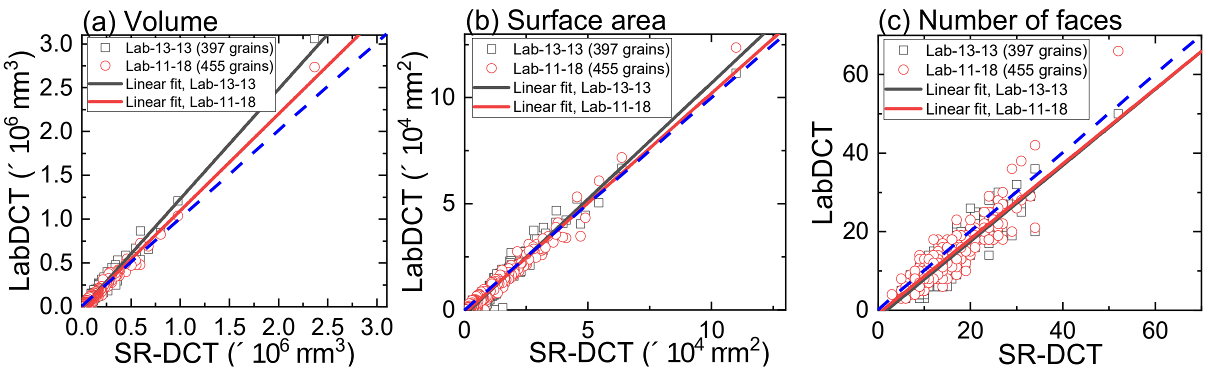

Figure S5 Comparison of (a) grain volume, (b) grain surface area and (c) number of grain faces for the one-to-one indexed grains between the Lab-13-13 or the Lab-11-18 and the SR-2.5 dataset. The solid lines show linear fits and the blue dashed lines indicate identical values between LabDCT and SR-DCT datasets. In (c) the black and red solid lines are almost overlapping.

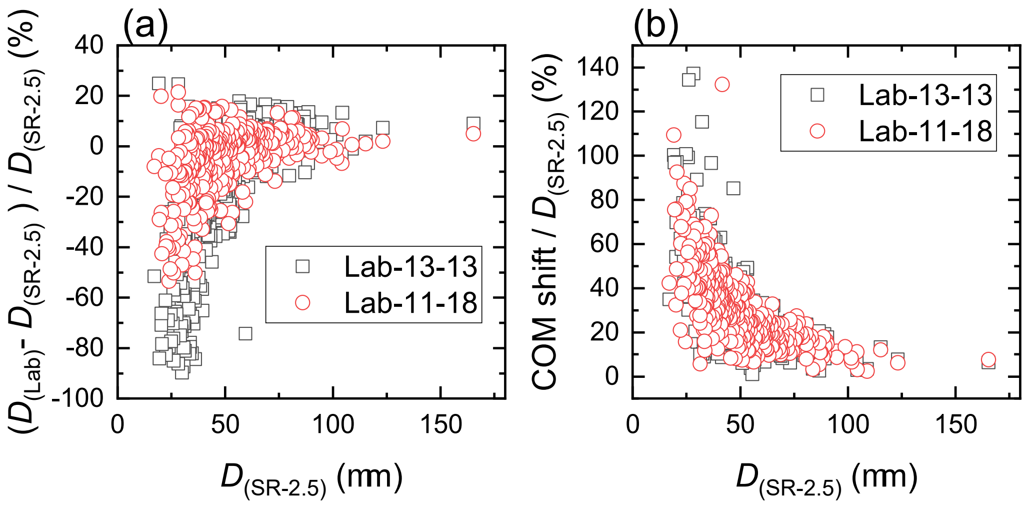

Figure S6 (a) Relative grain size difference and normalized shift of grain center-of-mass (COM) as a function of grain size for the 388 one-to-one common indexed grains. It shows that both grain size difference and COM shift are generally smaller for Lab-11-18 than Lab-13-13.

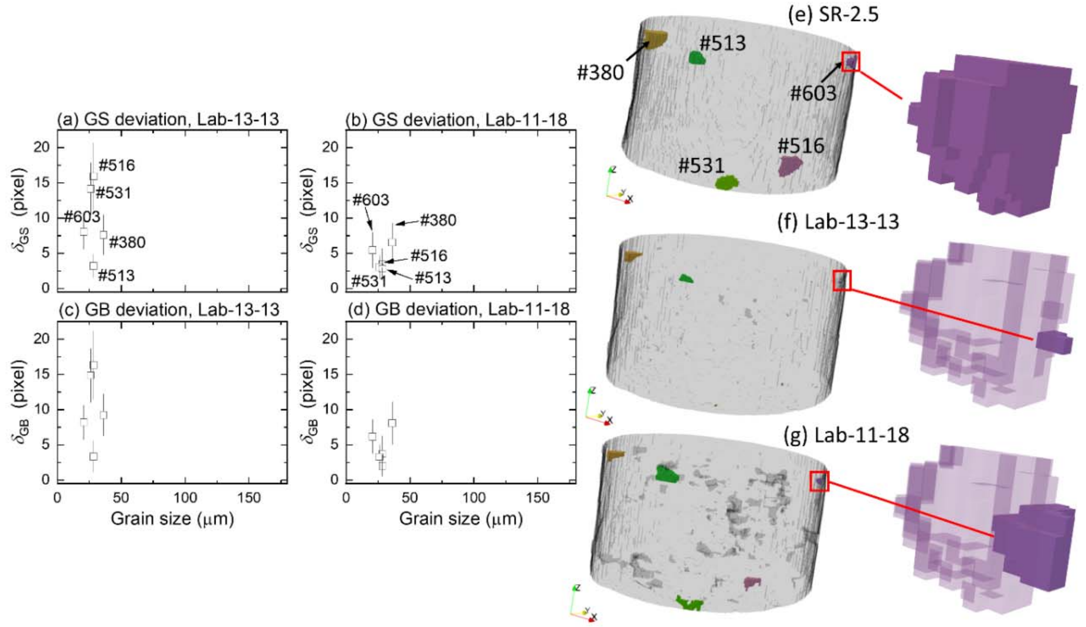

Figure S7 (a, b) Grain shape deviation δ_{GS} and (c, d) grain boundary deviation δ_{GB} of five relatively small grains in the Lab-13-13 and Lab-11-18 datasets. (e-g) Visualization of the five grains in SR-2.5, Lab-13-13 and Lab-11-18 datasets respectively. All the grains are located at the sample surface except grain #513. The corresponding zoom-in views of grain #603 are shown on the right side. The grain reconstructed from SR-2.5 is made semi-transparent in the zoom-in views of the LabDCT datasets.

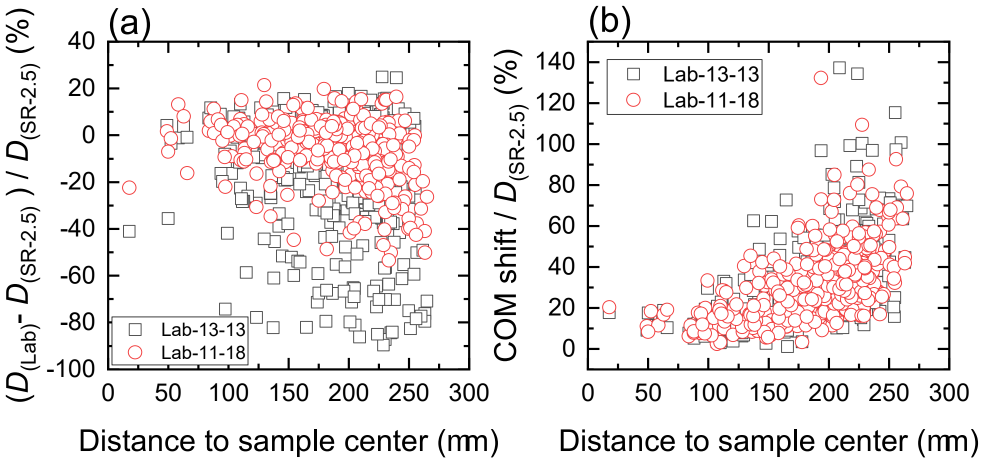

Figure S8 (a) Relative grain size difference and normalized shift of grain center-of-mass (COM) as a function of the distance between the grain center and the sample center for the 388 one-to-one common indexed grains. It shows that both grain size difference and COM shift correlate with the distance to the sample center for both LabDCT datasets.

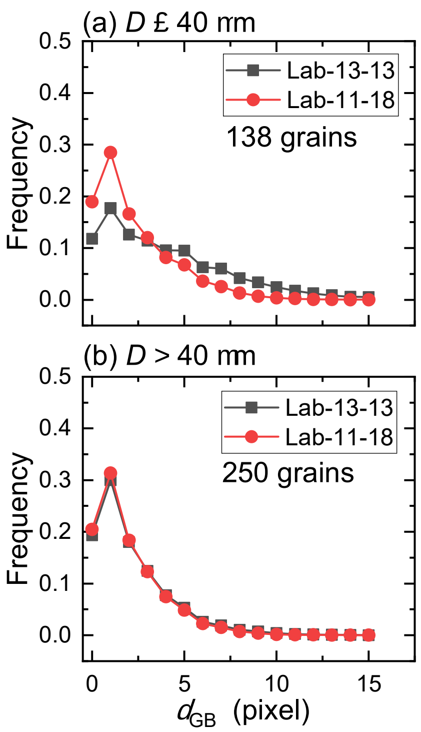

Figure S9 Statistics of GB deviations for 388 common one-to-one indexed grains in the LabDCT datasets grouped into two categories according to their sizes determined by SR-2.5. (a) 138 grains with $D \leq 40$ µm and (b) 250 grains with $D > 40$ µm.

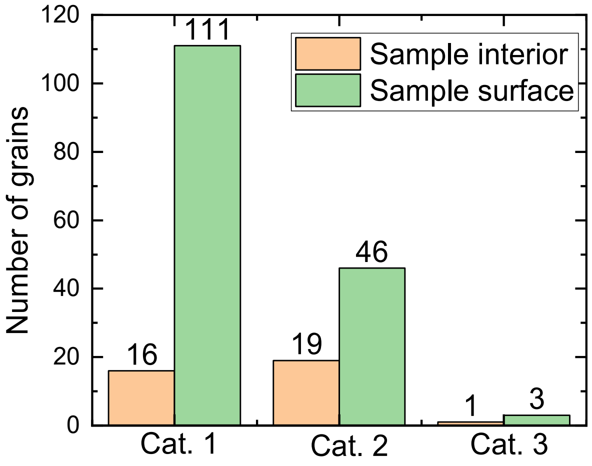

Figure S10 Number of false-negatively indexed grains classified in three categories (see the main text) determined separately according to their locations: in the sample interior or at sample surface.

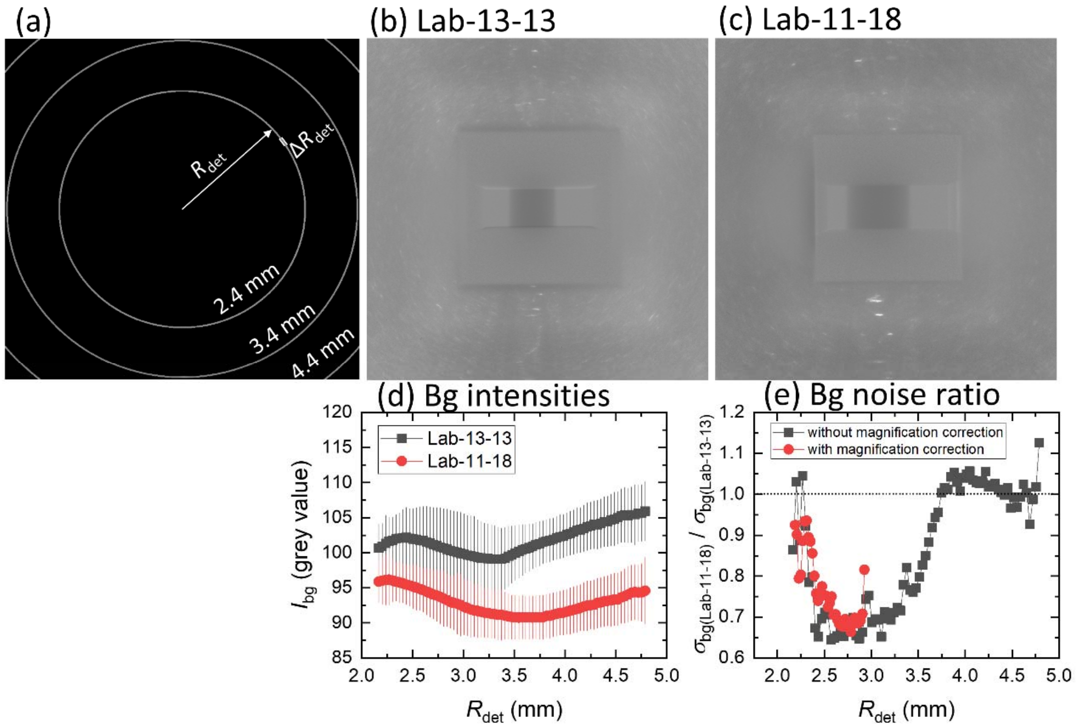

Figure S11 Background intensity (I_{bg}) and noise (expressed as standard deviation of background intensities, σ_{bg}) of each ring with a radial (physical) distance to the center of the detector (R_{det}) and a thickness of ΔR_{det} which is 10 pixels (*i.e.* 33.6 μm). (a) Illustration of three particular rings within which most of the spots are expected; Images containing mainly the background intensities which are used for this calculation and were obtained by rolling median processing over 11 sequential diffraction images for (b) Lab-13-13 and (c) Lab-11-18; (d) I_{bg} as a function of R_{det}; (e) The ratio of $\sigma_{bg(Lab\text{-}11\text{-}18)}$ to $\sigma_{bg(Lab\text{-}13\text{-}13)}$ as a function of R_{det} with and without the magnification correction, which accounts for the outward location of the same spot on the same detector as L_{sd} / L_{ss} increases from 1 to 1.64. The noise ratios for 2.4 mm < R_{det} < 3.4 mm are far smaller than 1, indicating that the background noise in Lab-11-18 is much smaller than that in Lab-13-13 for the region where most of the spots are located.

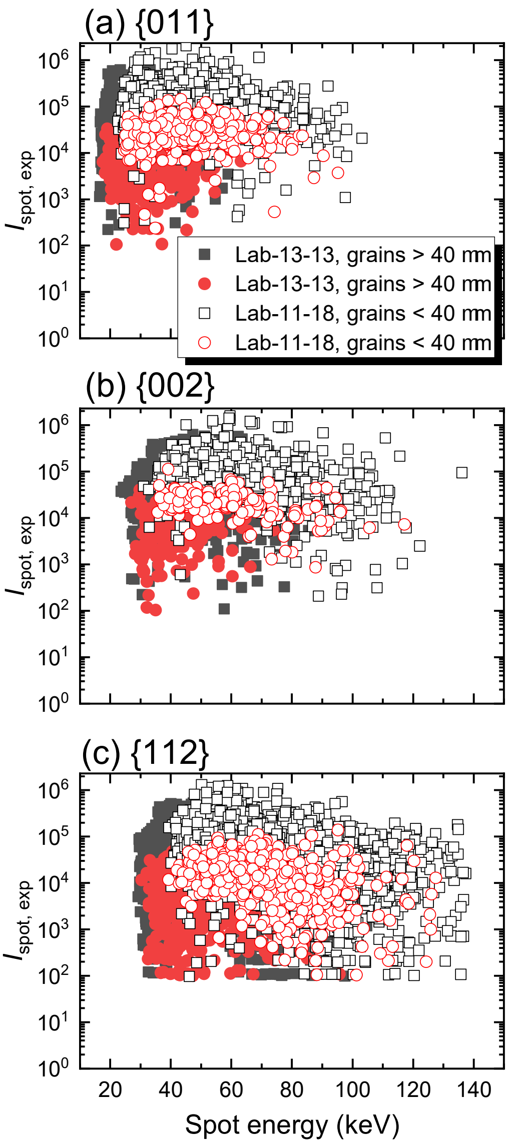

Figure S12 Integrated intensities of experimental spots, $I_{spot, exp}$, as a function of spot energy for (a) {011}, (b) {002} and (c) {112} families from grains subdivided into categories being larger or smaller than 40 µm in datasets of Lab-13-13 and Lab-11-18. The spot energy ranges are all changed towards higher energies for Lab-11-18 compared to Lab-13-13. The main reason is that with increasing L_{sd} low energy spots disappear (hitting outside the detector), while high energy spots appear (blocked by the beam stop in Lab-13-13). However, it should be noted that a slight decrease of L_{ss} from 13 to 11 mm causes some spots shifting their energies from a lower into a higher range. When this occurs for spot energies in the range 22 - 33 keV in Lab-13-13 shifting to 33 – 45 keV in Lab-11-18 (corresponding to an increase in detective quantum efficiency will occur. This explains the pronounced increase of $I_{spot, exp}$ observed for {011} as shown in (a).

Other supplementary tables:

Table S2 Summary of false negatively indexed grains identified according to their spatial locations.

	At sample surface				In sample interior			
LabDCT dataset	N	$< D >$, μm	σ_D, μm	f_V	N	$< D >$, μm	σ_D, μm	f_V
Lab-13-13	157	16.6	7.2	0.0147	35	24.4	6.2	0.0076
Lab-11-18	114	14.0	5.5	0.0059	17	20.7	5.3	0.0022

Table S3 Summary of false positively indexed grains identified according to their spatial locations.

	At sample surface				In sample interior			
LabDCT dataset	N	$< D >$, μm	σ_D, μm	f_V	N	$< D >$, μm	σ_D, μm	f_V
Lab-13-13	4	10.8	2.7	0.7×10^{-4}	3	8.0	5.0	0.4×10^{-4}
Lab-11-18	13	9.3	4.1	2.1×10^{-4}	6	9.9	4.2	1.1×10^{-4}

References

Lind, J. F. (2013). *In-situ high-energy diffraction microscopy study of zirconium under uni-axial tensile deformation*. Carnegie Mellon University.
